# Exploration of treatment burden during transition onto kidney replacement therapy: A longitudinal qualitative study

**DOI:** 10.1371/journal.pone.0360290

**Published:** 2026-10-12

**Authors:** Catrin Jones, Benjamin Edgar, Karen Stevenson, Bhautesh Jani, Patrick Mark, Katie Gallacher

**Affiliations:** 1 School of Health and Wellbeing, University of Glasgow, Glasgow, Scotland; 2 School of Cardiovascular and Metabolic Health, University of Glasgow, Glasgow, Scotland; 3 Department of Renal Surgery, NHS Greater Glasgow and Clyde, Glasgow, Scotland; Phramongkutklao College of Medicine, THAILAND

## Abstract

**Background:**

People with kidney disease who need to transition onto kidney replacement therapy (KRT) face a life-changing diagnosis. This process involves complex decisions about treatment and a high volume of healthcare work. Little work has been done looking at KRT transition through the lens of treatment burden. This study aims to explore how treatment burden evolves during KRT transition in a population recruited from a large nephrology and kidney transplant unit in Glasgow, Scotland.

**Methods and findings:**

Serial in-depth qualitative interviews were conducted with 30 participants across up to three time points, generating 76 interviews. We used a framework analysis informed by Normalisation Process Theory (NPT), Theory of Patient Capacity (TPC) and Burden of Treatment Theory (BoTT) to explore the interaction between workload, capacity, and the system. Five main themes affecting treatment burden arose: engaging personal and social resources to navigate KRT transition, being able to meet the challenges, preparing to enact the healthcare workload, undertaking KRT transition related workload, and the evolution of burden over time.

**Conclusion:**

Transitioning onto KRT is a process that entails a high volume of healthcare workload and significant treatment burden. Experiences of treatment burden are influenced by the nature of the work required; the patient’s capacity including physical, psychological, financial and cognitive factors; the capacity of their social network; and the functioning of the healthcare system. Particular focus should be placed on understanding the impact of the healthcare system on experiences of burden, as it represents a potentially modifiable driver of treatment burden that clinicians and healthcare policy-makers can directly influence.

## Introduction

Patients who need to start kidney replacement therapy (KRT) face a considerable increase in their healthcare workload. Treatment burden is the impact of healthcare workload on patient wellbeing [1]. Three middle range theories have been developed to better conceptualise workload, patient capacity and the burden of treatment. Normalisation Process Theory (NPT) describes the processes through which patients ‘normalise’ healthcare workload, meaning the process through which patients incorporate new complex healthcare tasks into everyday life. It has four main domains: Coherence, which is the work of understanding and making sense of healthcare work; Cognitive Participation, which is the work of planning and enrolling others; Collective Action, which is the work done by the patient and their network to ensure the work occurs; and Reflexive Monitoring, which is the work of monitoring, evaluating and adapting their execution of the work [2]. The Theory of Patient Capacity (TPC) was developed to describe the factors that influence the ability to undertake healthcare work. It categorises patients’ capacity as five different elements: Biography, which refers to the ability to navigate changes in their social roles as a result of chronic illness; Resources, which are the material and personal resources including financial, physical health, cognitive, medical and health system knowledge, transport and time resource that a patient can mobilise; Environment, which is the influence of the home, community, and healthcare environment within which the patient is trying to undertake the work; Realisation of work, which is the effect of patients’ experiences of performing healthcare work on their capacity to undertake further work; and Social, which is the effect of the patients’ social network on their capacity [3].

Burden of Treatment Theory (BoTT) conceptualises the relationship between the healthcare workload delegated to patients to undertake, their capacity to undertake it, and the healthcare system. It describes the interaction between workload and capacity in terms of ‘agency’ which is the patient’s ability to engage with health problems and with others, ‘relationality’ which are the social networks through which agency can be expressed and distributed, ‘control’ which are the things that healthcare systems do to determine the structure and content of services, and ‘opportunity’ defined as the availability of services in different areas at different times. It also describes the interactions between the patient, their social network and the system as they mobilise their capacity and enact healthcare tasks. The theory postulates that the demands placed on patients to enact healthcare work can be experienced as treatment burdens, and patients become overburdened if their workload exceeds their capacity to meet it [4].

We have previously created taxonomies of workload and capacity during transition onto KRT in a qualitative systematic review [5]. This review identified that transitioning onto KRT was a period of high healthcare workload and the factors that impacted patient capacity, but was limited in its ability to understand the relationship between capacity and workload, the evolution of burden over time or the effect of the healthcare system. The aim of this study is to describe the interaction of healthcare workload, patient capacity and treatment burden during the process of transition onto KRT and how it evolves over time.

## Methods

Ethical approval was granted by the Proportionate Review Sub-Committee of the North West Preston Research Ethics Committee, IRAS project ID 322865, REC reference 23/NW/0267. Written consent was obtained from all participants.

### Study design

#### Recruitment of participants.

This study is a longitudinal qualitative study underpinned by framework analysis. The population of interest are adults over the age of 18 approaching the need to start KRT for the first time in a single large health board area in Scotland. Participants were selected using a purposive stratified sampling approach; stratified by age, gender, starting modality, planned or unplanned start, and ethnicity. The aim of the sampling strategy was to capture a diverse range of experiences of KRT transition. Potential participants were identified from nephrology clinics, vascular access clinics, peritoneal dialysis (PD) clinics, transplant assessment clinics and inpatient admissions and approached in person. Inclusion and exclusion criteria are detailed in Table 1. Information power was used to guide our sample size [6]. Five factors were considered: study aim, sample specificity, use of theory, quality of dialogue, and analysis strategy. We also considered the risk of clinical deterioration or death of participants during the study period due to the nature of advanced kidney disease. The study has a narrow and focused aim, and although we needed to capture a range of modalities the population of interest was highly specific. The longitudinal design with rich, in-depth interviews over time gave us confidence that the quality of the dialogue was likely to be high, and the framework approach grounded in previous work gave us confidence that the analysis strategy was likely to have a high yield. We therefore estimated a sample size between 25–40 followed up for up to 12 months would provide sufficient information power whilst remaining feasible, and we stopped recruiting at 30 participants. We elected to use information power to inform our sample size rather than thematic saturation as information power considers whether the data collected is sufficiently rich to answer the research question, rather than focus on the absence of new themes emerging. Thematic saturation has been criticised for being ambiguous in what truly represents saturation, and for encouraging coarse coding and broad themes in order to achieve so-called data saturation rather than deep reflexive and interpretative engagement with the data [7].

**Table 1:** Inclusion and exclusion criteria for recruitment.

Inclusion	Exclusion
Adults aged 18 or over	Children aged under 18
Capacity to consent	Already established on KRT (>1 month) prior to recruitment
Starting any modality of KRT for the first time with an anticipated start date within next 6 months	Anticipated recovery of kidney function, e.g., KRT in ICU in context of critical illness
Sufficient English language skills to be able to understand consent process and participate in interviews without the aid of a translator	

### Data collection

Semi-structured interviews lasting approximately 30−45 minutes were conducted either in person or online depending on the participant’s preference. Participants were interviewed up to three times: once prior to the initiation of KRT (or peri-initiation in unplanned starts) to capture the burden of preparatory workload, once 6 weeks to 3 months after starting to capture the burden of transition, and once 6–12 month after initiation to capture evolution of burden over time. Separate semi-structured topic guides were developed for each of the three interviews, which were informed by NPT and TPC and developed with input from a patient-public engagement group and clinicians. Interviews were conducted between 1^st^ January 2024–30^th^ June 2025. Interviews were recorded on an encrypted digital recorder and transcribed verbatim. Field notes were made after the interview. Transcripts were not returned to participants for comment, and there was no further participant checking. The majority of interviews were conducted solely with the researcher and the participant, but a minority elected to have a caregiver with them. Questions were directed at the participant but spontaneous contributions and clarifications from the caregiver were included for analysis.

### Analysis

Data analysis was facilitated by Nvivo 14. The five stages of framework analysis (Familiarisation, Identifying a Thematic Framework, Indexing, Charting and Mapping) were adhered to [8]. Following familiarisation, line by line coding of all transcripts using a coding framework developed using NPT to describe workload domains and TPC to describe patient capacity domains was conducted by the first author, with regular coding clinics with the senior author to enhance the reliability of the coding. Analytic disagreements between the two authors were rare, and were resolved through discussion. The initial coding framework was developed informed by a published NPT coding manual [9] and a prior systematic review [5] and applied deductively. Any concept falling outwith the NPT and TPC were coded separately, and specific separate codes were developed iteratively to characterise changes to workload, capacity and burden over time. There were no workload or capacity concepts that fell outwith the initial coding frame, but all transcripts had themes relating to the evolution of burden over time which were coded inductively. We charted the output from the first order coding and developed taxonomies of workload and of capacity. We subsequently applied the BoTT domains of mobilising capacity, expressing capacity, mobilizing for delegated tasks and enacting delegated tasks to the coding framework along with an additional higher order theme that pertained to the evolution of burden over time. This higher order analysis of how workload and capacity interact in the context of a healthcare system and the evolution of treatment burden over time during KRT transition informed our five themes. There were no negative or divergent cases that required active consideration during the analysis process.

### Research team and reflexivity

The primary researcher who conducted all the interviews (CJ) is a female General Surgery and Renal Transplant Registrar in their mid-thirties with prior qualitative research experience and training. She holds a MBChB and a MSc. At the time of conducting the research they were employed in a non-clinical role as a doctoral research fellow, but had worked clinically in the unit previously. They had no prior relationship with the participants, and participants were aware that this was research conducted as part of a PhD. They have no personal or family experience of KRT, so approached this study with a professional interest and substantial prior knowledge of the clinical side of KRT transition but less insight into the patient experience. Having a clinical background may have had both a positive and negative effect on the interviews and the analysis. Familiarity with kidney disease and its treatment could mean that patients felt at ease discussing complexities and nuances in their treatment journey confident that they would be understood. It could also aid with the analysis due to a prior deep understanding of the subject matter, and mean that the researcher is less likely to misunderstand or misinterpret the participants clinical course than a researcher without a relevant clinical background. However, it is possible that having a clinical background in KRT care could have negatively influenced the interviews and their analysis. Participants may have been more guarded in criticising the service or the staff. When analysing the data, it is possible that independent professional relationships with the clinicians involved and prior understanding of the strength and weaknesses of the KRT services from previously working in the unit may have sub-consciously coloured the interpretation of the data. Conscious efforts were made to centre the patient’s voice and remain data driven at all points but it is inevitable that a clinical background and lack of lived experience influences the understanding of the data.

## Results

30 patients were recruited and completed the initial interview [Table 2], of which 26 completed a second interview (4 did not progress to start KRT within the study timescale), and 20 completed a third interview (2 died, 2 started KRT too late to reach 3^rd^ interview timepoint within study period, 1 withdrew consent for participation and 1 was lost to follow up), giving a total of 76 interviews included in the analysis. Of the 30 patients recruited, 8 changed modalities (Fig 1).

**Table 2:** Participant characteristics by starting modality.

	HD(n = 15)	PD (n = 7)	KTx (n = 8)
Sex Male Female	7 8	3 4	4 4
Age 18-44 45-64 65-74 75+	5 5 4 1	3 1 2 1	0 4 4 0
Modality change PD/HD to KTx PD⇔HD/ HD⇔PD	2 1	2 3	0 0
Ethnicity White Asian Black Other	13 2 0 0	6 1 0 0	8 0 0 0

*HD-haemodialysis; PD- Peritoneal dialysis; KTx- Kidney Transplant*

**Fig 1:**
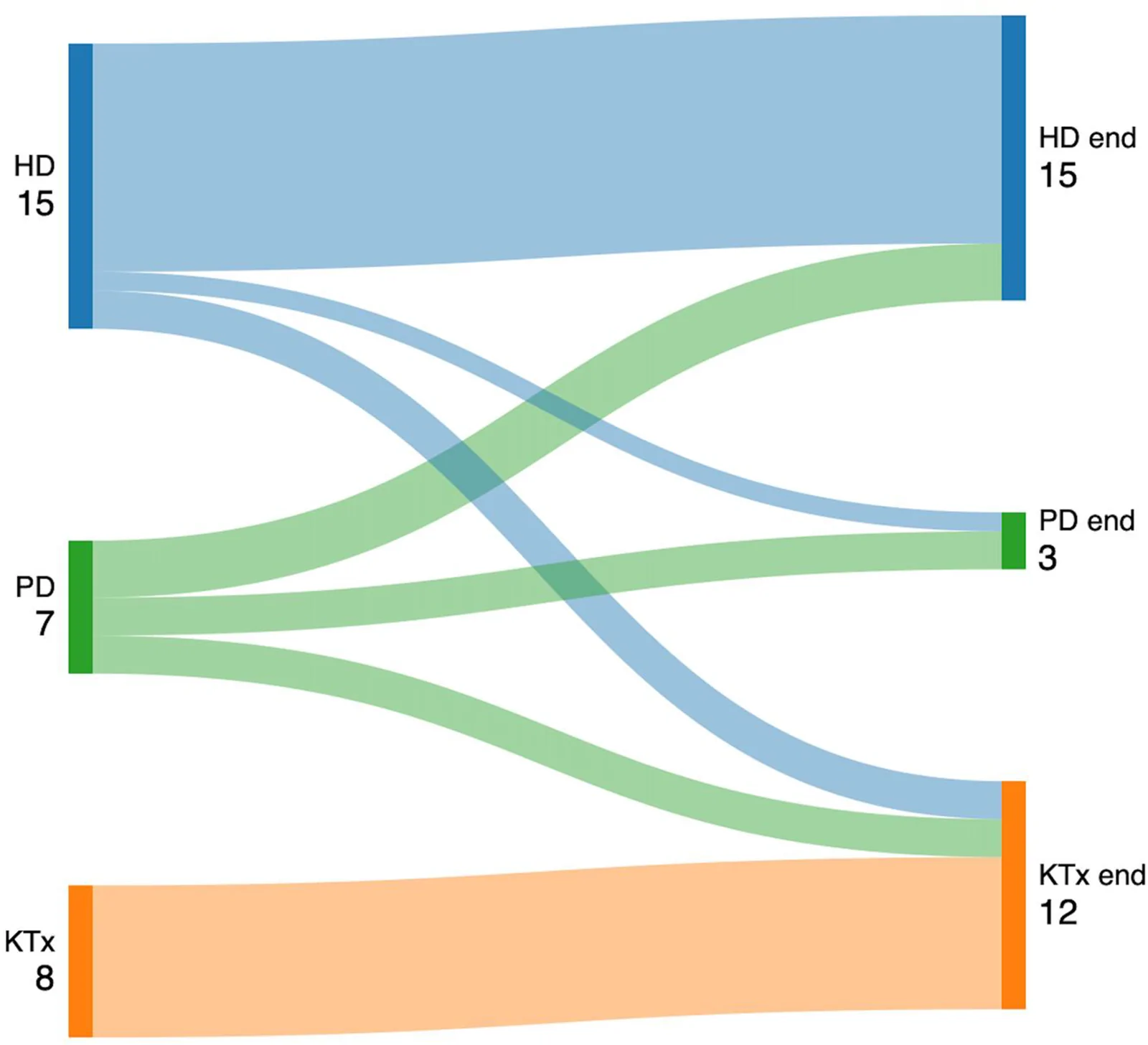
Modality changes over 12 months. Sankey diagram demonstrating changes in modality participants experienced over the study period demonstrating changes from initial starting modality to KRT modality at 12 months.

When looking at how treatment burden is experienced through a BoTT lens five main themes arose: four distinct theme (themes 1–4), and one cross-cutting (theme 5). Different elements of the taxonomy of workload and the taxonomy of capacity were mapped onto the BoTT themes as detailed in Table 3.

**Table 3:** Workload and capacity taxonomy domains mapped on to burden of treatment themes.

Workload taxonomy domain	Capacity taxonomy domain
**Theme 1: Engaging personal and social resources to navigate KRT transition (*Mobilising Capacity)***	
Appropriateness of allocation of work	Biographical disruption
Effect of interaction with healthcare or governmental services	Biographical adaptation
Recruiting others	Literacy
Understanding what KRT means for their social network.	Medical and health system knowledge
	Physical health
	Psychological resilience
	Capacity building environments
	Negative experiences of the care environment
	Ability of social network to accept condition and effects
	Personal ability to fulfil social role
**Theme 2: Being able to meet the challenges of KRT transition (*Expressing Capacity)***	
Recruiting others to help	Financial resources
	Literacy
	Medical and health system knowledge
	Access to paid support services
	Physical health
	Psychological resilience
	Self-efficacy
	Time
	Transport
	Ability of their social network to accept condition and effects
	Social relationship with their healthcare team
**Theme 3: Preparing to meet the healthcare workload of KRT transition (*Mobilizing for delegated tasks)***	
Working out how and why life on KRT will be different	Experiences of the realisation of work
Understanding what KRT means for their social network	
Understanding what KRT will mean for them as an individual	
Finding value in KRT	
Driving forward work required to start KRT	
Recruiting others to help	
Deciding that their chosen modality of KRT is the right option for them	
Maintaining key interventions in daily life	
Monitoring the impact of KRT	
Evaluating effect of KRT on family/social network	
Reflecting on how they feel about KRT	
**Theme 4: Undertaking KRT transition related work (*Enacting delegated tasks)***	
Doing the work required by KRT	Experiences of the realisation of work
Effect on relationships and the confidence people have in each other	Capacity building environments
Appropriateness of the allocation of work	Negative experiences of care
Effect of interaction with healthcare or governmental services on patients	Provision of instrumental support

### Theme 1: Engaging personal and social resources to navigate KRT transition

The interaction between personal agency, capacity held within family and wider social networks, and the opportunities and constraints on access to care mediated by the structures and provisions of the healthcare system had a profound impact on participants’ experiences of navigating the process of transitioning onto KRT (Fig 2).

**Fig 2:**
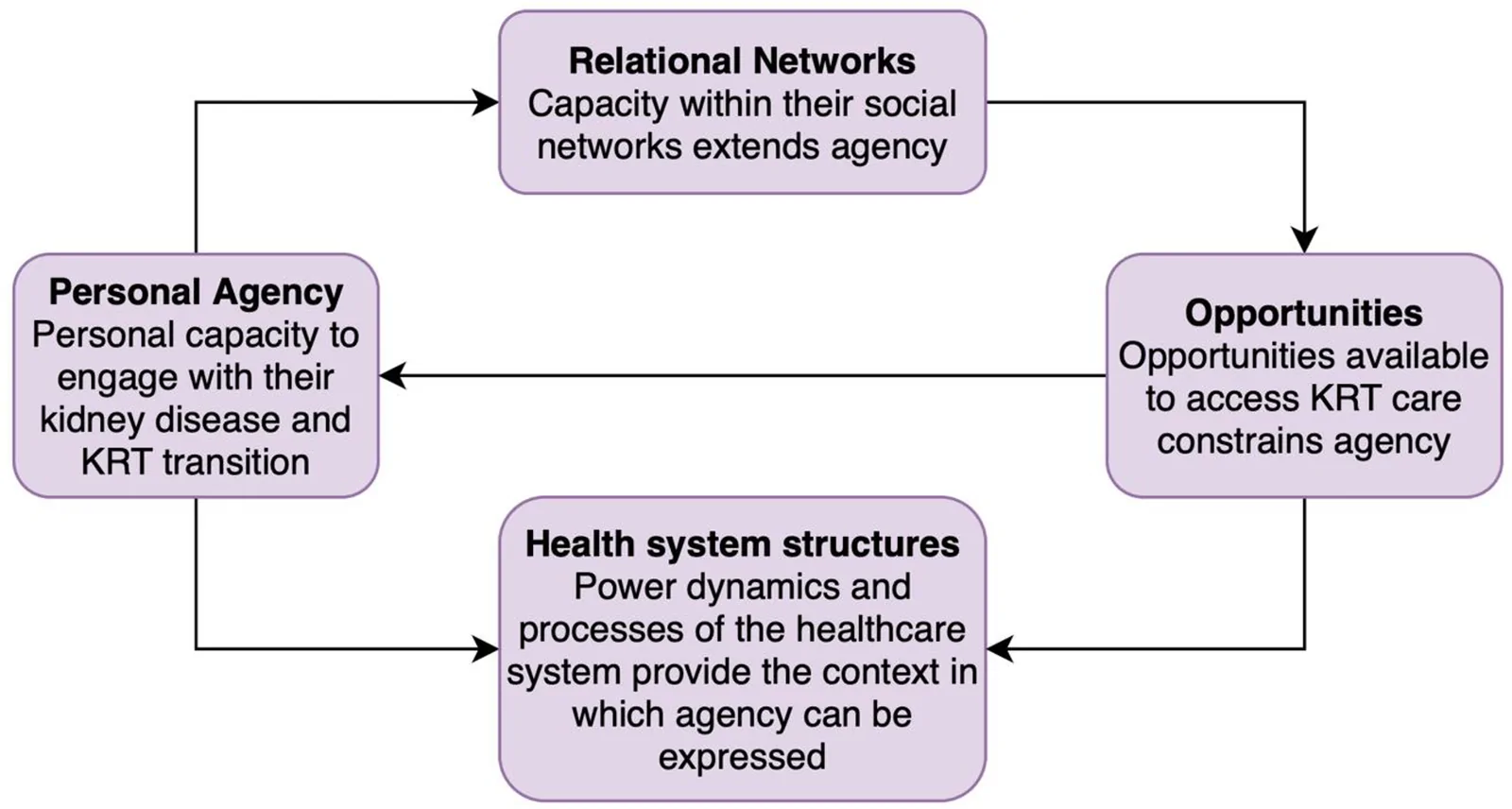
Engaging personal and social resources to navigate KRT transition. Flow diagram demonstrating the interaction between personal and social resources during KRT transition and their impact of the generation of treatment burden.

Personal agency was heavily influenced by psychological and physical factors. All participants described sadness and apprehension at receiving the news that they would imminently require KRT, and all found the nature of the decisions they were required to make and the prospect of the healthcare work they would be required to undertake daunting.

The time between first diagnosis of kidney disease and the need to start KRT was an important influence on agency during this period. Some participants had known for many years that they were likely to require KRT and therefore had opportunity to prepare both psychologically and practically, maximising their potential agency. For some, their kidney disease progressed rapidly and they were required to make decisions regarding KRT urgently at a time where they were confronted with a life-changing diagnosis as well as a significant symptom burden constraining both their capacity and their opportunity to pursue certain avenues such as pre-emptive transplantation. Biographical disruption, which is the impact caused by the deviation from expectations of life caused by illness, had a profound impact on some participants, especially those that were younger or had developed advanced CKD unexpectedly.

*It was completely out of left field, ‘cause I was still very active, I surfed the weekend before I got diagnosed. Erm, it was a tough, tough pill to swallow even more so because everyone kept saying you don’t fit the regular patient…[I was] very taken aback and obviously thrown my life into turmoil at this stage* PDM2

Relational networks, especially close family and friends, were an important source of support and therefore capacity for many participants. Relational networks act to extend individual agency, and a key part of enacting the complex work of KRT was building and maintaining relational networks that could be relied upon. However, the potential negative impact of their condition on their loved ones was an area of grave concern for many, as well as the changing relationship dynamics from care-giver to care-receiver and from provider to dependent. Live donor transplantation brought this to the fore, with a unique emotional workload processing what it means to have a loved one donate a kidney, and balancing their concern and love for them against their desire for a transplant.

*The most difficult part of this illness… you don’t want anybody to give you the kidney and you pray for somebody to offer you a kidney, at the same time. It’s very difficult because your natural instincts are saying, do everything to save yourself, to save your life, to survive. And your heart, and emotions, and attachment to [potential live donors] because these are usually the closest family members or friends, the dearest people. So, you don’t want them to suffer. Er, and at the same time, you put them in this position, and it’s hard.* KTF2

The quality and accessibility of services influenced participants’ ability to mobilise their capacity. Many described deriving great value from the input of specialist nurses as they prepared to transition onto KRT, with home visits and extended appointments enhancing their understanding of their condition and increasing their confidence in the system. Others described difficulty in getting information from clinicians and feeling disempowered and frightened in an indifferent system. Some described feeling like they needed to do extensive independent preparatory work prior to consultations to successfully self-advocate.

*There’s been times where I think, not through deliberate action... there’s not been the communication or the explanation of things for me. And when I've asked, not everyone has been forthcoming with their answer. So, I've actually done an awful lot of reading myself. So, before it came to the choice of what my options were, I had read up on it, and I knew what my options were... So that, when I come into the doctor, I'm not saying to the doctor, what is this? I'm saying to the doctor, I know what this is, but what about that.* HDF3

On the other hand, some described long-term holistic relationships with clinicians enhancing their agency, for example in supporting the emotional burden of accepting a living donation.

*I was refusing a live donor…I wouldn’t take a kidney from my son…I couldn’t countenance putting him through it…you know naturally through evolution I’m there to protect him and he’s not there to protect me. [The nephrologist] got me in contact with somebody who’d actually been through transplant, it just helped me to speak to someone who’d been through it. Who wasn’t ashamed or embarrassed to say, look, I took something from my boy because it was going to help me live better.* KTM2

### Theme 2: Being able to meet the challenges of KRT transition

Participants’ ability to function in the different contexts that KRT transition work is undertaken, their social skills securing co-operation, and their ability to absorb adversity heavily influenced their ability to meet the challenges of KRT transition (Fig 3).

**Fig 3:**
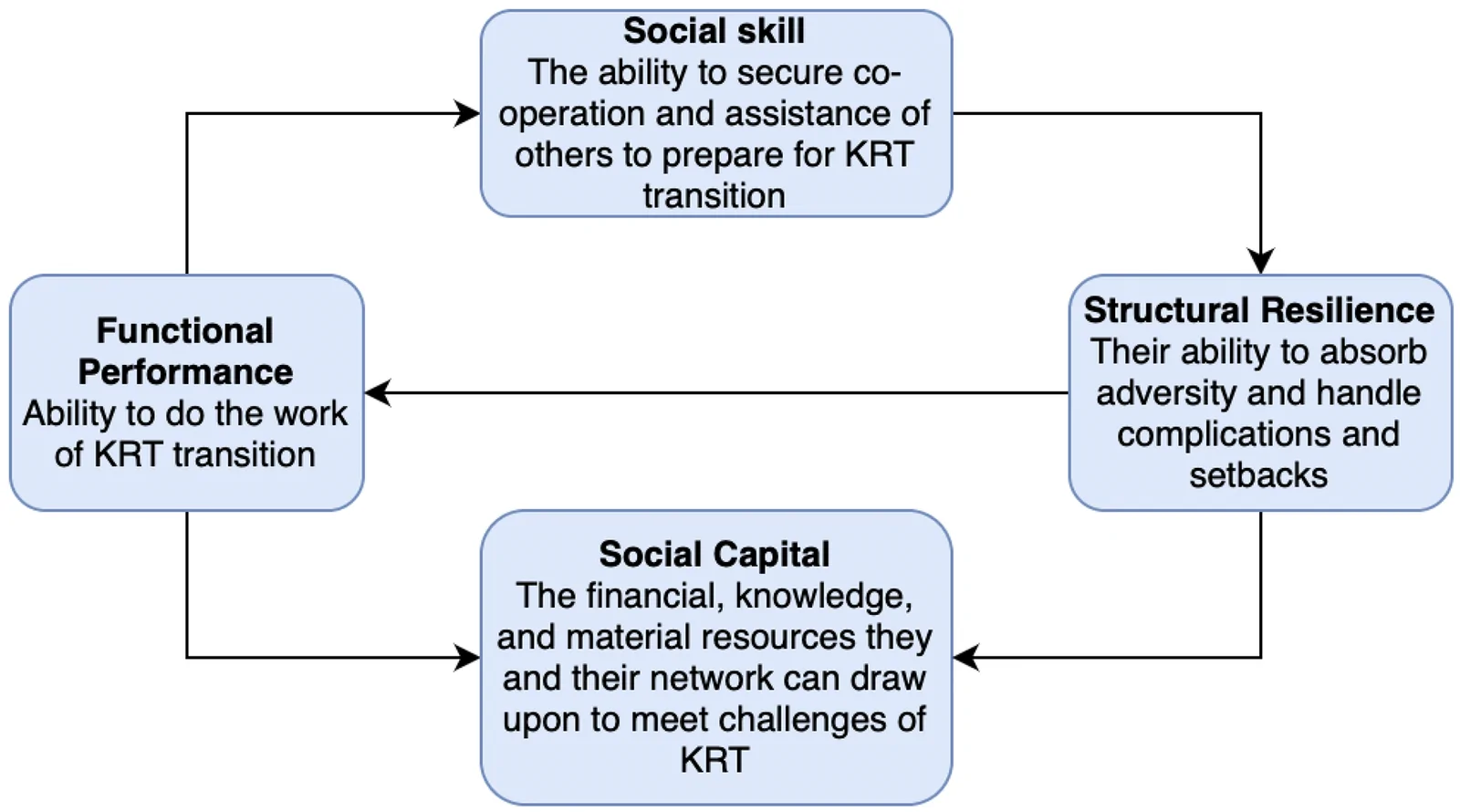
Being able to meet the challenges of KRT transition. Flow diagram demonstrating the interaction between different aspects of patient capacity during transition onto KRT.

The financial, educational, cognitive, and psychological resources participants had to draw upon as well as their current health status and physical capabilities all contributed to their functional performance. Those with prior understanding of how the healthcare system worked and had prior medical knowledge were better equipped to navigate KRT transition. Financial concerns were a considerable source of burden especially for younger participants who were unable to work due to their illness, with the practical impact of a sudden loss of income compounded by the biographical disruption caused by the loss of career and social role. Personality was also important, with several participants crediting their psychological resilience for helping them cope with the impact of KRT transition.

A close-knit family network provided not only material assistance but comfort and a sense of purpose. Participants often described family and friends assisting materially with transport and activities of daily living such cooking and cleaning, and being sources of information and support. Those without a robust, mobilizable social network had noticeably weaker structural resilience, often highly vulnerable to single point failure. For example, those without family and friends that could be called upon were reliant on hospital transport and if that failed then they would miss important appointments despite considerable efforts on their part to attend.

*Today, I was due to go to an access nurse interview. And, erm, somehow, there was a miscommunication, the person that came to pick me up couldn’t take wheelchair patients. So, they had to cancel it, and get somebody to come over from somewhere else to pick me up, to bring me in for dialysis. But it meant I missed my appointment for the access clinic.* HDF3

Those who had recently migrated were particularly vulnerable, often describing social isolation on top of a lack of system’s knowledge and additional work managing the concerns of, and for, their family back home.

*Because of me, everybody worried. My parents in India worried…my mum is too much worried… it’s very hard because they live there, I live here, so they can’t come here. I can’t go there because of my dialysis gonna start soon… From childhood my parents help me to grow… I too much depended on my parents. But when I came here I work, I try to become to stay alone and manage myself. But now again, I come to that point I need my parents.* HDM8

The mobilizability of a person’s social network was a key determinant of the feasibility of pre-emptive live donor transplantation. Some described having multiple potential donors coming forward proactively, their donor using their health system knowledge and self-efficacy to drive forward the assessment process, and their wider social network rallying around to support the process. Others described not knowing anyone suitable for live donation, being unsure how to discuss live donation with family, and difficulty navigating the assessment process especially in the context of international donation.

*I spoke to my brothers, and they all decided that they would go forward for the test… I put it on the family group chat...I just said that I'm getting to the stage where I either need to get a kidney, or go on dialysis. They’ve mentioned about the living donor scheme. I said, now if any of yous are interested, let me know. Er, and three of them got back to me straightaway, and I gave them the details.* KTM4

Healthcare staff formed part of the mobilizable social network. Having consistent long-term doctor-patient relationships especially with nephrology was highly valued by participants. System processes that did not facilitate continuity of care inhibited the recruitability of trusted clinicians into participants’ network, inhibiting capacity.

*Other people talk to you and they say about your renal team, but it’s like I don’t have a renal ‘team’. When you go to clinics…its whatever doctor’s ready comes out and gets your sheet and calls you in. And I find that frustrating. I suppose it’s the way that the NHS works ‘cause the GPs is the same, but it’s like you feel they never really know your set up…I’ve seen one doctor twice but everyone else only ever once. So there’s no connection.* HDF7

### Theme 3: Preparing to meet the healthcare workload of KRT transition

Preparing to meet the workload of KRT encompasses the work of sense-making, building and maintaining social networks that can help with this work, and constantly monitoring and adapting as their situation evolves over time (Fig 4).

**Fig 4:**
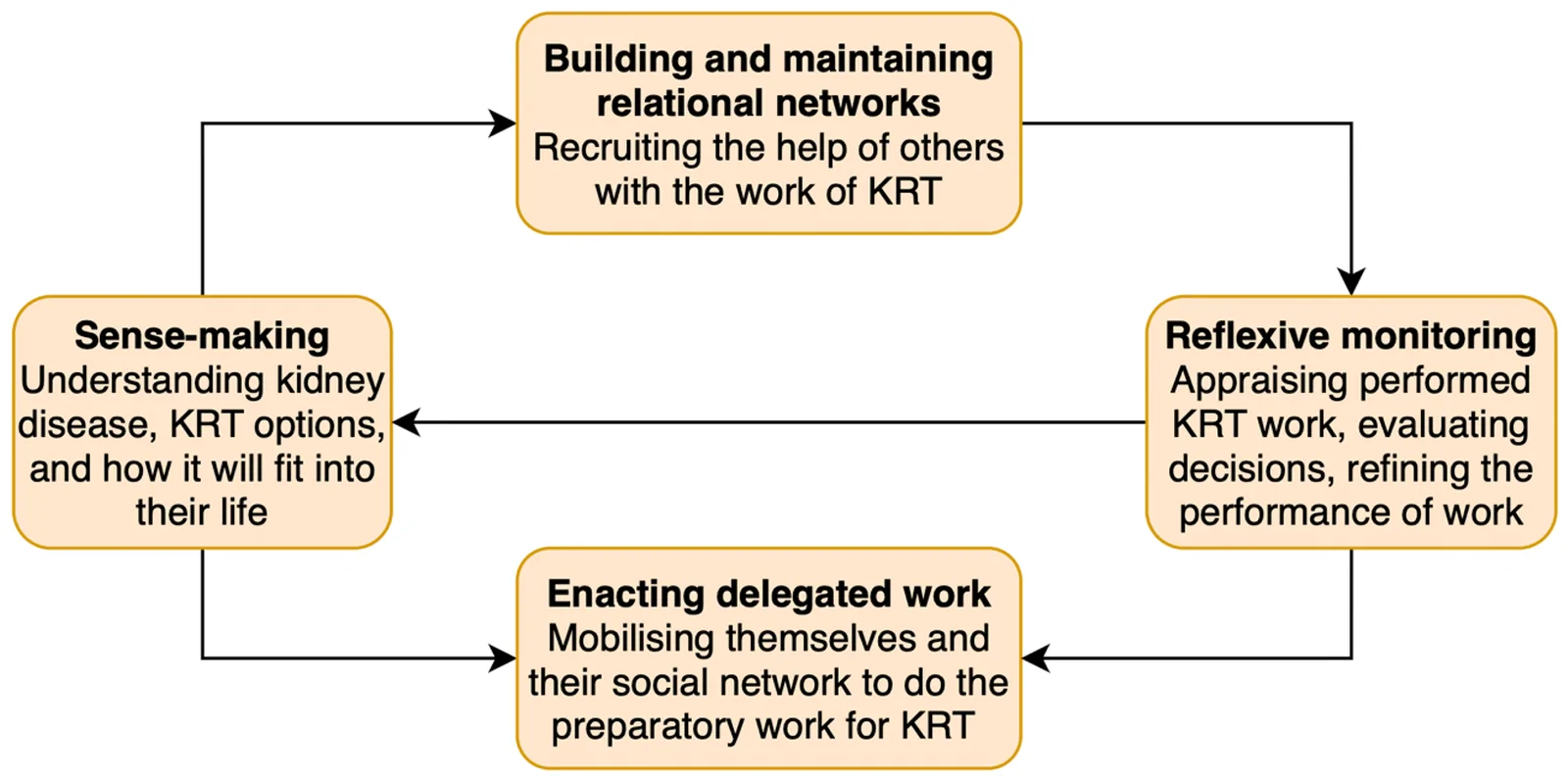
Preparing to meet the healthcare workload of KRT transition. Flow diagram demonstrating the interaction between sense-making, relational, operational, and reflexive workload during transition onto KRT.

Sense-making around KRT initiation focused on understanding what different KRT modalities mean in terms of the resources required to do the work, the opportunities available, and how these fit with their wishes and expectations of life. This was particularly prominent in decisions around home therapies (PD and home HD), where two main aspects determined participant’s decisions. Firstly, practical and logistical considerations were considered: whether they had the necessary physical space, manual dexterity, and social support to undertake home dialysis. Secondly, psychological and emotional consideration predominated, especially considerations around undertaking medical work in their home. Some found the delegated responsibility and freedom offered by home therapies liberating, and some found the concept intrusive and frightening, preferring the compartmentalisation and safety offered by in-centre dialysis.

*The girls came out to the house to discuss potential types of dialysis, but when they talked about the other types I felt as a blind person they were not remotely practical…we felt very strongly that hospital was the right way to go… [I’m] desperately keen to get a transplant… so the plan is to get hospital haemodialysis until a transplant becomes available* HDM2

Deciding on the best KRT option for them depended on understanding how various options would fit into the context of their life and how things could develop over time, for example the likelihood of a future transplant. Some participants also put a great deal of importance on understanding what had superior outcomes.

*We’ll be doing home haemodialysis. So, at some point, I'll start the training to learn how to do that. [My wife] will get involved. They’ll then come and set up a dialysis station at home for me, and then I can dialyse at home. Because my understanding is, is that long term, the outcome for people who dialyse at home is slightly better than people who come to hospital to dialyse. And then, obviously, I then wait for a suitable kidney to become available for a transplant.* HDM1

Evaluating and reconfiguring the work of KRT encompasses all the work of monitoring, identifying issues and finding solutions, refining their workload, and reflecting on their experiences. Patients who felt a significant benefit from KRT felt increasing confidence in their choices and their ability to do the work. Negative experiences could undermine confidence in their ability to manage their care. For example, participants who had been successfully performing PD at home but subsequently went on to develop complications reported lower confidence in their ability to undertake the work.

*I think [PD peritonitis] might have [reduced my confidence] for a wee bit at the beginning, because I was trying to figure out how it had happened... It’s made me wary of is every time I change the dressing…It made us worry that it was something that we’d done, that we hadn’t been meticulous to our cleaning or washed our hands or we’d touched something.* PDF3

Several participants reported distress in the face of multiple setbacks and the realisation that life on KRT and the path to transplant can be uncertain and vulnerable to complications. Participants who had been transplanted had a much lower workload by their third interview, and a growing normalisation of their remaining workload into everyday life. But several had a persistent psychological burden. Reaching a point of greater stability often provided the opportunity to reflect on how difficult KRT transition had been. It also brought to the fore the reality of a new worse normal: that transplant offered a better quality of life than a life on dialysis but would never return them to life as it was before they developed advanced CKD. The burden of gratitude and the burden of the expectation to be a ‘worthy’ transplant recipient could be considerable: the perception of a societal expectation to be grateful of the ‘gift’ of transplantation even when they were still burdened by significant symptoms and grieving their lost life.

*I was quite low for a while, it was consuming me, really… [the doctors] are really happy, and everything’s going well with my kidney, which is amazing. But me, myself, still feel tired, and all my bones are sore, and there’s things that are pulling me down…You know, I have had this lifeline thrown at me, and if I didn’t have it, then the outcome could be completely different. And I think that’s quite a sobering thought as well, you know, it’s been a necessary life-saving procedure… but there are all these life changing things that are quite hard to process. I'm not back out doing what I did before, I've got limits… it’s really, really difficult, because although I'm so grateful, and everybody is like, oh my goodness, what a gift your sister has given you and I'm going, ‘I know, it’s just incredible’. It’s not really connecting with me, you know, that way, it’s like, I'm, I'm saying it, I'm saying all the right things, but underneath it all I'm like, eugh.* KTF4

Participants who had undertaken the sense-making and mobilising work for a certain treatment option only to have to change to another were especially burdened. This was particularly marked in participants that perceived that this deviation from what they had worked towards was as a result of the system not working as it should. Feeling that the system had control over their lives but they had no control over the system was highly attritional to their perception of their own agency, undermined their relation with clinicians, and placed greater stress on their social network.

*My dad’s my living donor. I didn’t want to do dialysis so I held on as long as I could. But then I think it got to a point that my body was too weak, that even if surgery was to go ahead in the next few days, that the doctors felt that I probably wouldn’t make it through the surgery… they basically pulled the plug and said, enough is enough. I feel that dialysis has made me feel worse… I said in the beginning I didn’t want it, and I was determined not to have it. Erm, and then I felt as though everything was just, kind of, dictated by the doctors*. HDF8

### Theme 4: Enacting KRT transition work

Implementing the work of KRT encompasses the experience of enacting the practical work of KRT such as implementing dietary changes; attending clinics, dialysis, and procedures; undertaking the work of home therapies; rehabilitating after transplant surgery; managing medication regimens; seeking care for deteriorations; and liaising with employers and the welfare system for necessary accommodations (Fig 5).

**Fig 5:**
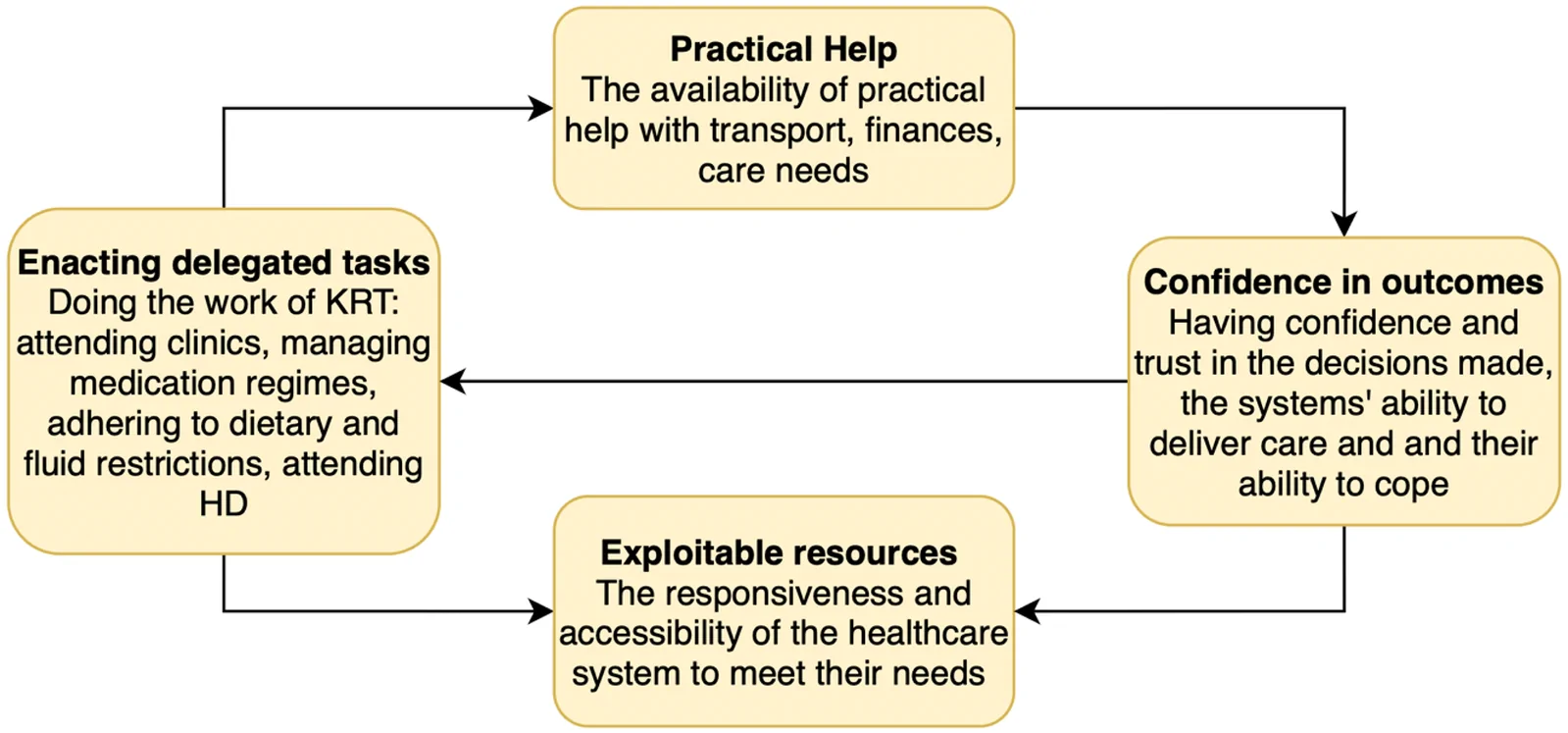
Enacting KRT transition work. Flow diagram demonstrating the interaction between the experiences of enacting KRT transition workload and its effects on treatment burden,.

Participants described significant workload that encroached on every element of their lives. The fluid restriction on dialysis was often described as being particularly onerous, requiring constant vigilance and disruption to long embedded social routines.

The most important determinants of participants’ experiences of implementing the work of KRT were the practical help and the local exploitable resources available to them, especially the accessibility and responsiveness of the healthcare system. The interface between different components of the system were a particular area of disjointed care where considerable onus was placed on the patient to bridge gaps in provision and communication. Some patients described being asked to attend appointments that they had no means to travel to or arrange tests in the community that community services couldn’t provide, sometimes resulting in serious harm. Participants also described experiencing discrepancies between the care that they expected and the care they received, with safety nets that they had been assured existed failing to materialise.

*When I phoned up and said, I'm really not very well, getting sent to my local GP for my bloods, I didn’t think was particularly helpful, especially since the hospital knows I've got rotten veins…But they wanted me to go through the process of going to the GP, it not working, and then I could go back to the hospital. And that, that felt pretty rough…it’s quite difficult as well, because when I've been in hospital before, various doctors have said to me, if you feel unwell for anything at all, phone up and come straight in, and we’ll get you sorted. And the last two or three times where I've really been very unwell, I've phoned up, and they’ve not taken me in you know, they’ve asked me to do something else…each time, it’s kind of, had to get to a crunch point before I've ended up in hospital. And I'm not sure that’s always been necessary.* HDF3

Managing the supply of vital medications that were recommended in hospital, prescribed by primary care, and dispensed by community pharmacies was a constant battle for several participants. This was exacerbated by the increasing use of digital platforms that excluded those with lower levels of digital literacy and increased the complexity and inefficiency of ordering systems.

*I don’t understand [why it’s so difficult to get medications] because they say that you can do it on the website by creating an NHS account, but we have an NHS account because we can see all the results on Patient Access. But for some reason, because we’ve created an account with the NHS through Patient Access, it doesn’t allow us to use the email ordering system with our surgery so we have to phone it in. So we phone it in…and a week later we’ll go, more often than not, the pharmacy hasn’t received it, and we phone up the surgery and they say we never got your request. Luckily, my pharmacist knows me now … she’ll give me an emergency script to keep me covered. But that shouldn’t be happening. I think I’m gonna have to change my practice purely because they can’t get my scripts right or get it out on time.* HDM1

The physical geography of healthcare provision was a source of burden for some patients, especially in the context of the hub and spoke transplant model where patients are required to travel to the central hospital for frequent follow up for the first 12 months post-transplant. Other services were also fragmented, and many participants described needing to attend different appointments at different hospitals, often encountering heavy traffic and difficulty parking. Most participants did not feel well enough to take public transport and the provision of hospital transport was unreliable and not universal, therefore arranging travel to appointments was a source of considerable burden.

The complexity, inflexibility, and facelessness of the system could be highly burdensome to patients. Experiences of expending considerable energy mobilising themselves and their network to enact work only to be frustrated by an inflexible and unresponsive system were deleterious to patient capacity. Participants were entirely dependent on the system to provide them with life-saving KRT and therefore the control the system could enact over participants’ lives and the effects that the limitation in opportunities could have upon them was considerable.

*There’s nothing I can do. There’s certain things within my control and stuff that is outside my control so I accept it and carry on…It’s a big machine with so many moving parts, very difficult to influence which way it goes...There’s definitely parts of the process which could have been expedited and been illogical, it seems…Sometimes you feel like you’re in the dark…it was impossible to get information on [transplant dates], we went round in circles for about a week. So it’s an instance of being frustrated again and also the…the process itself just seems like it’s out of antiquity, like it needs to be updated and realise that people are going through quite stressful times and trying to organise their life as well… it’s really frustrating.* PDM2

### Theme 5: Evolution of burden over time

When evaluating the evolution of burden over time in longitudinal interviews, the interaction between individual, social network, and healthcare system factors and the progress and outcomes of the healthcare work undertaken heavily influenced ongoing burden (Fig 6).

**Fig 6:**
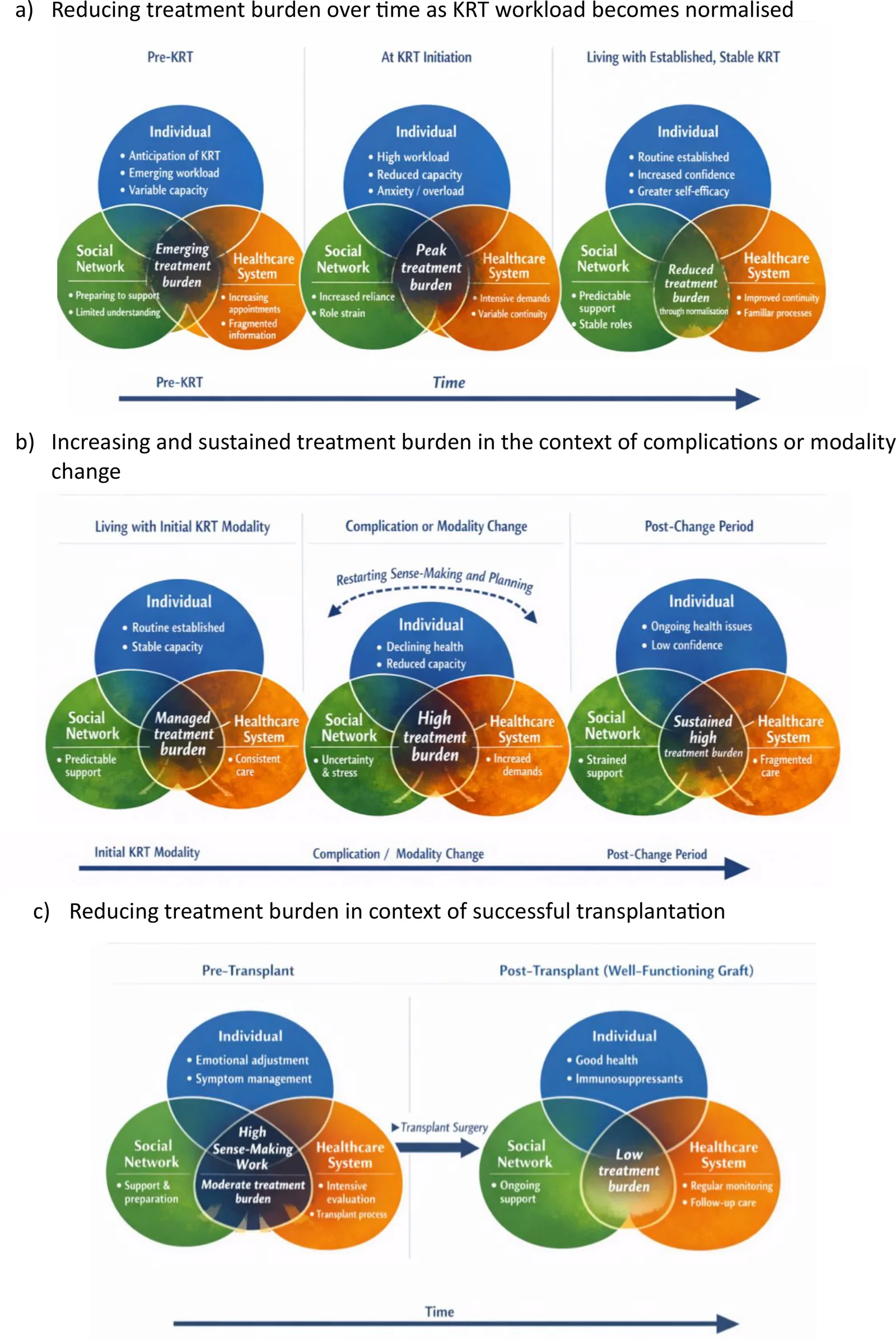
Variations in the evolution of treatment burden during KRT transition. A) Diagram of treatment burden reducing over time as KRT workload becomes normalised into everyday life B) Diagram of increasing and sustained treatment burden over time in the context of complications or modality change. C) Diagram of reducing treatment burden over time in context of successful transplantation.

We have further illustrated this with three clinical vignettes (Table 4). Relative stability was key to participants’ ability to incorporate KRT into daily life and ‘normalise’ the work. Those who had normalised KRT workload and incorporated it into their routine were less burdened and had greater confidence in their ability to undertake healthcare workload, their social network’s ability to support, and the healthcare system.

**Table 4:** Clinical Vignettes illustrating the evolution of burden over time.

Participant	Pre- KRT	Post initiation	Follow up at 6–12 months
KTF4	Preparing for pre-emptive live donor transplantation burdensome. Two main sources of burden: 1. Significant sense-making burden in both processing the implications of CKD, the need for transplantation, and the emotional workload of accepting a donation from a loved one. 2. Navigating the live donor process. Difficulty getting information about progress of work up necessitating multiple phone calls to self-advocate. Delays resulted in deteriorating physical health and an unsuccessful and unwanted attempt at initiating PD.	Treatment burden evolved away from sense-making and system navigation to dealing with practicalities of recovering from a major operation. Burden driven by: 1. Understanding and implementing new medication regime 2. Wound management 3. Co-ordinating transport for regular clinic attendances 4. Managing side-effects of medication 5. Physical recovery and rehabilitation 6. Understanding and adapting to immunosuppression: food hygiene and dietary considerations, avoiding crowded places, sun safety	Notably less burdened. Physical health much improved, medication side-effects largely resolved, and taking immunosuppressants normalised into daily life. Able to take on far more social roles, making plans for holidays, quality of life much higher. Remaining burden predominantly driven by biographical disruption: accepting that this is the ‘new normal’ and that physical health may never be good enough to return fully to prior function (especially full time work). Burdened by emotional aspects of being a transplant recipient: feeling indebted, burden of gratitude, guilt for having negative feelings about life when a loved one had donated to her.
PDM2	Rapid development of kidney disease: biographical disruption, sense-making work around future implications, huge unexpected disruption to professional and personal life. Decision to pursue PD as bridge to live donor transplantation: sense-making and co-ordinating work for PD initiation and starting LDTx process. Lots of information to seek out and process, but also hope that a fairly rapid live donor transplant will improve things soon. Self-directed research around nutrition optimisation. Intensive training to prepare to perform PD independently empowering and not overwhelming: able to do something to help himself.	Healthcare workload high in establishing PD routine in the home, but well supported and slowly gaining confidence. Unforeseen work such as managing storage of supplies and waste disposal of PD consumables requiring troubleshooting and refining routine. Considerable emotional and practical workload navigating LDTx process, with growing frustration and mistrust of the integrity of system processes. Financial and professional implications of extended period unable to work starting to become more pressing.	PD failure resulting in unplanned start on HD and hospitalisation. LDTx process protracted with several potential transplants falling through for a combination of recipient, donor, and system factors. Very frustrated and burdened by failure to progress to LDTx despite being highly proactive. Biographical disruption exacerbated by delays to transplantation. Further loss of autonomy with move to ICHD. System factors major exacerbator of burden, especially poor communication and poor processes. Feels very disempowered. New sense-making work with HD, new vascular access decision making and care, new stricter fluid and dietary restrictions. Capacity to undertake this notably diminished from earlier: having previously applied considerable effort and resources to facilitate PD and KTx which had so far proven unsuccessful confidence in self and system diminished.
HDM4	Decline in kidney function slow then fast, therefore although kidney disease longstanding KRT start somewhat unexpected. Critically ill at KRT start therefore physical and cognitive resources limited. Biographical work in understanding and accepting that he has a life threatening illness. Sense-making work relatively limited: too unwell to either have many options or to engage with nuances of decision making process. Keen to follow medical team advice and to delegate responsibility for decision making.	Physical symptoms much improved from acute crisis that required hospitalisation and start on dialysis, but learning to manage new symptoms such as fatigue and itch that fluctuate with dialysis timetable. Now feels well enough to engage with sense-making around dietary and fluid restrictions. Co-ordinating family help with activities of daily living such as shopping and cleaning. Dialysis start initially in hospital but finally settled into regular dialysis slot at preferred dialysis centre.	Settled in his dialysis unit, with weekly routine established. Developed relationships with staff members and other regular patients- now part of social network. Perfected dialysis set up: with preferred snacks, entertainment downloaded on his tablet, chair set up, vascular access care, transport all optimised. Making plans for a holiday abroad with dialysis visits.

*Ah it’s just…pretty easy. I come off [the PD] in the morning and that’s me. I don’t need to do anything else with it until night time, and then what I do normally is I go up about half eight/nine o'clock at night and I’ll set the machine up so it’s just sitting ready just to press the button for the priming, so that’s it sitting ready and then I go to my bed maybe half eleven/twelve, press the button and start draining it off and that’s you, away you go, and then it’s finished about half eight in the morning. In terms of socialising and, kind of, hobbies and stuff I'm still managing to play football. I've played for the last four weeks… I'm going to play golf this Saturday for the first time in a year.* PDM1

Those who experienced complications or needed to change modalities had difficulty normalising their work as it kept changing, therefore they had to restart sense-making and planning for a different modality as well as dealing with workload generated from the complication and the resulting decrease in capacity from worse physical health. This workload directly and indirectly increased burden, by both increasing the healthcare workload required and undermining confidence in their choices, the healthcare system and their ability to undertake healthcare work.

*The PD was horrific. I didn’t want dialysis, I didn’t want it in my house that was hard for me, because this is your safe space… I felt that the, the hospital was coming in here and in my mind, it was making me sick, which is really silly, because I know I've been really sick. But I didn’t allow myself to think I was…I don’t really do hospitals very well. So that was hard, going in for the operation to get the wee tube put in. And then, I had, I had problems with it from day one, I was in excruciating pain and just horrendous side-effects with my bowel, it was awful. Then obviously, it didn’t work. And it not working, et cetera, then getting the peritonitis, which was really terrifying… I was uncontrollably shaking, and in excruciating pain...Then getting the news that the transplant was a couple of weeks away which is incredible and I should be really, really happy and delighted…but I've just been through hell and here I am again, it was just all so much*… *What bothers me with that is, it, I feel it could have been avoided had the transplant gone ahead when it was all good to go… that whole episode wouldn’t have happened.* KTF4

All participants described high workload and experiences of treatment burden but the severity and evolution of the burden differed between modalities. Kidney transplant patients often described higher sense-making and planning workload initially, especially the emotional work of coming to terms with accepting a transplant and managing the symptoms of advanced CKD, but a considerably lower burden compared to HD and PD patients when they were interviewed when they were living with a well-functioning transplant kidney. With PD and HD, workload differed depending on whether dialysis was their intended definitive KRT or whether it was a bridging KRT whilst they awaited transplantation. Patients who started on PD or HD and were subsequently transplanted all reported a reduction in their treatment burden, but the period before the transplant when simultaneously managing the workload of starting KRT on a dialysis modality and the workload of preparing for transplantation was especially burdensome. For HD patients the stability of their vascular access for dialysis had a significant impact on burden, with pre-emptive stable vascular access highly protective. Vascular access complications could be a source of considerable burden. For PD patients, all reported a high initial workload learning how to perform PD and preparing their homes and family life to undertake the work. This workload reduced over time as PD became normalised into their daily routine. Once established on PD, treatment burden was primarily driven by experiences of complications such as PD peritonitis or PD inadequacy rather than by performing routine PD.

## Discussion

### Summary of findings

In this work we have explored how treatment burdens are experienced through engaging personal and social resources to navigate KRT transition, creating the ability to meet the challenges of transition, preparing to enact the healthcare workload and undertaking KRT transition related workload, and how this evolves over time. This provides a comprehensive and novel understanding of treatment burden during KRT transition.

The study of the evolution of burden over time in this work is novel and has not been explored in other work. We identified three main treatment burden patterns over time (Fig 6). Firstly, the general trend is for healthcare workload and treatment burden to increase rapidly during the period immediately preceding KRT initiation, peak at the time of initiation, and subsequently reduce as the workload becomes normalised and the burden subsides. The nature of the workload also evolves, transitioning from initially sense-making and preparatory co-ordinating work before starting, to more task based enacting and co-ordinating work at the time of initiation, and eventually to more refining and reflexive work when established on KRT. Secondly, for some patients, treatment burden remains high and they do not experience a transition to greater stability and lower burden. This is predominantly driven by instability through complications, modality changes, and poor systemic processes. This instability means that participants could not progress to the refining and reflecting stage as they were perpetually required to understand and make decisions about new treatment avenues and co-ordinate their social network to enact new tasks in response to events, leading to sustained high burden. Thirdly, successful transplantation can have a transformative effect on burden, offering the possibility of a much lower healthcare workload and increased capacity due to improved physical health.

A key finding in our study is the importance of the accessibility and responsiveness of healthcare systems. In our study three key areas emerged where poor system functioning increased treatment burden: lack of continuity of care within renal services, poor interface between different system components, and divergence between expectations of care and lived experiences. Participants’ relational network was key to enhancing capacity in our study, with friends and family providing support and assistance at all stages of the transition journey. What is also clear in our study is that starting KRT has a profound impact on families, with a large amount of new work required of a participants’ loved ones without much support to help them adapt.

### How it fits with other work

Other work supports the finding that kidney failure simultaneously reduces physical and cognitive capacity whilst increasing workload leaving patients highly vulnerable to becoming overburdened, and that a patient’s social network is a vital enhancer of agency resulting in patients with a weak network being vulnerable to harm [10–12].

Studies of the impact of biographical disruption on experiences of kidney disease align with our findings that the impact of the loss of role, physical abilities, expectations of life and social functioning can be profound and impair a patient’s capacity to engage in healthcare workload [13,14]. Studies of the impact of KRT transition on families found that family members of KRT patients reported negative experiences in becoming a carer for the patient, dealing with adverse psychological responses including sadness and anxiety, burden from treatment delivery and logistics, and burden from dealing with increased symptoms in the patient [15,16].

Health system issue including continuity of care and frequency of healthcare encounters have been identified in other work as the most important source of treatment burden in advanced CKD [17]. The concept of minimally disruptive medicine recognises that a substantial burden is modifiable and results from how healthcare is organised and delivered, and that non-compliance should be understood not as poor patient behaviour but as a symptom of poor system delivery [18–22].

There is a large body of evidence that health related quality of life improves after transplantation [23,24]. Health related quality of life is different to treatment burden in that it mostly captures the effect of illness rather than the effect of treatment, however there is considerable overlap between improving symptomatology and the physical, cognitive and psychological health domains of patient capacity and possible reduction in health related workload.

Little work has been done studying patient capacity in the context of advanced CKD but studies of patient activation, defined as a patient’s knowledge, skill and ability to manage their own health, in advanced CKD have shown that patients with higher levels of activation were more able to engage with shared decision making and adhere to treatment [25,26], and lower activation is associated with worse health related quality of life [27]. There is some evidence that interventions aimed at improving patient activation can improve self-management and self-efficacy in advanced CKD [28]. Patient activation is a narrower, more individualistic concept than capacity but there is significant overlap between the two concepts and it would be reasonable to assume that activation is a proxy for some domains of capacity.

### Implications for clinical practice, policy, and further research

Our findings suggest that improving the access to transplantation for those who are potentially eligible may represent the most potent intervention to reduce burden at the level of the individual. This adds to the body of evidence that kidney transplantation is associated with both an improvement in quality of life from pre-transplant and a superior quality of life to HD or PD, and that seeking to achieve early transplantation offers the best outcomes for suitable patients [23,24,29]. There are significant systemic limitations to this as there is a national shortage of deceased donor organs and not all patients have potential live donors [30]. Improving both rates of transplantation and access to pre-emptive transplant would require local and national work. On a national level, efforts to increase donor organ availability and utilisation could increase the availability of decreased donor transplant and reduce the waiting time. On a local level, improving accessibility to transplant assessment could improve access to transplant especially in driving forward the requisite investigations to facilitate listing. Prioritising timely pre-emptive vascular access and focusing on improving procedure selection could also significantly reduce the workload experienced by patients. This is wholly compatible with the Kidney Disease Outcomes Quality Initiative (KDOQI) ‘right patient, right procedure, right time’ approach to dialysis vascular access [31].

Whilst treatment burden is multifactorial and influenced by many factors relating to the patient, their social network, and the healthcare system, understanding the impact of health system function on burden is important as it is a potentially modifiable factor that clinicians and policy makers can directly impact, whereas other factors that are the product of the wider socioeconomic functioning of society are more intractable. KRT involves a significant disruption in patients’ lives, however prioritising innovations that facilitate better continuity of care with key clinicians and the fostering of lasting doctor-patient relationships; ensuring seamless communication between different components of the system; and building capacity that would facilitate the system to be flexible around the needs of the patient rather than requiring the patient to be flexible around the needs of the system could significantly reduce burden.

It has been hypothesised that capacity could be assessed and coached by clinicians by using a structured discussion aid to explore the different domains of capacity and identify areas that could be improved [32,33]. Whether a KRT specific capacity discussion aid could help clinicians to identify areas of low capacity and tailor interventions to help is a potential avenue of further research. Interventions could be developed to bolster KRT patients’ peer-to-peer network with other KRT patients, with formal peer-to-peer support groups found to improve patient engagement with care, treatment adherence, and self-efficacy in patients with kidney failure [34]. Interventions could also be developed that improve the support offered to care-givers and the wider relational network and could therefore reduce the treatment burden experienced by patients by enhancing the capacity of their social network.

### Limitations

Our study had several limitations. Firstly, it is a single centre study conducted in the West of Scotland which limits its generalisability to other cultural contexts. We were unable to offer translation services due to financial constraints and therefore were only able to recruit those whose English was sufficient to conduct interviews without support. This is a significant limitation as it does not capture the treatment burden generated by navigating a healthcare service with limited proficiency in the dominant local language. Language barriers could represent a major direct and indirect source of treatment burden, and this has not been addressed by this work. The ethnic diversity in the study population (90% White, 10% Asian) is representative of the ethnic diversity of the wider patient population served by the unit, but is not representative of the ethnic diversity of the renal population in the UK more widely which again limits the generalisability of the study.

The heterogeneity of the study population represents both a strength and a weakness. The study population represents different modalities and 8 out of 30 participants experience a modality change. This could be seen as a strength as it reflects the real life experiences of KRT transition: patients rarely fit into neat fixed categories but experience complications, delays, successful transplantations, and many other changes to their clinical and life circumstance and this range of experiences are reflected in the data. However, the heterogeneity of the population has meant that some subgroups are small and therefore caution should be exercised in drawing too definitive a conclusion from the findings. There was some loss to follow up, with only 20 of the 30 participants completing all three interviews and 4 only undertaking one. The most common reason for incomplete follow-up was slower than expected progression of CKD in those who’s intended first KRT modality was HD, resulting in either not starting KRT at all or starting with insufficient time for the third interview to be completed within the study period. Failing to fully capture the experiences of those with a more indolent illness course may mean that especially in our HD population we have disproportionately captured the experiences of those who have had a more rapid progression of their illness and quicker starts, and therefore different experiences of burden.

During the recruitment process we did not collect detailed screening, eligibility, or refusal numbers. The reason for this is that our epistemological stance for this work is interpretivist and that has informed our purposive sampling strategy. We aimed to recruit a rich sample that was representative of the range of experiences rather than a probability based statistically representative sample as would be sought in studies with a positivist approach. However, the absence of these data limits the assessment of recruitment flow or potential selection bias.

## Conclusion

Transition onto KRT is a time of profound change, with a rapid and substantial increase in healthcare workload at a time when their capacity to do the work can be severely reduced. Most patients experience high levels of treatment burden. This paper provides a detailed and novel exploration of the drivers of treatment burden during KRT transition and how it evolves over time. A key finding is that healthcare system processes are a prominent contributor to the experience of burden, and represent a potentially modifiable driver of treatment burden in this population. Developing treatment burden informed care for KRT transition could represent a potent way to reduce the burden experienced by patients. Secondly, for eligible patients, successful kidney transplantation could represent the treatment pathway that offers the lowest workload and burden, and interventions that improve access to transplantation are key to reducing treatment burden.

## Supporting information

S1 File COREQ checklist. (DOCX)

## Abbreviations

BoTT: Burden of Treatment Theory
HD: Haemodialysis
KDOQI: Kidney Disease Outcomes Quality Initiative
KRT: Kidney Replacement Therapy
KTx: Kidney Transplant
NPT: Normalisation Process Theory
PD: Peritoneal Dialysis
TPC: Theory of Patient Capacity
