# Supplementary material for: Exploration of treatment burden during transition onto kidney replacement therapy: A longitudinal qualitative study
Source: PLoS One. 2026 Oct 12;21(10):e0360290. doi: 10.1371/journal.pone.0360290 (PMC13657324; doi:10.1371/journal.pone.0360290)
Supplement: Supplementary file 3 [file pone.0360290.s005.docx]

School of Health and Wellbeing, University of Glasgow

Clarice Pears Building

90 Byres Road

Glasgow

G12 8TB

02/08/2026

Dear Editor,

We are pleased to submit our amended manuscript ‘Exploration of treatment burden during transition onto kidney replacement therapy: a longitudinal qualitative study’ for consideration for publication as an original research article in PLOS One.

We would like to thank the academic editor for their thoughtful comments, and have made the following alterations to the manuscript as outlined below.

1. *The abstract would be strengthened by explicitly stating the longitudinal nature of the study and the total number of interviews conducted. For example, the Methods and Findings section could state that 30 participants were interviewed across up to three timepoints, generating 76 interviews. This would better highlight one of the study’s key strengths.*

Thank you for your suggestion. Line 21 has now been amended to read “Serial in-depth qualitative interviews were conducted with 30 participants across up to three time points, generating 76 interviews.”.

2. *The manuscript now provides a useful explanation of information power. However, because some readers may expect discussion of thematic saturation, it would be helpful to state explicitly that information power, rather than thematic saturation, was used to guide sample sufficiency. This would avoid ambiguity and align the methods more clearly with contemporary qualitative methodology.*

Thank you for your comment. We have added the following passage at lines 102-108 to expand on why information power was chosen as our approach to sample sufficiency:

“We elected to use information power to inform our sample size rather than thematic saturation as information power considers whether the data collected is sufficiently rich to answer the research question, rather than focus on the absence of new themes emerging. Thematic saturation has been criticised for being ambiguous in what truly represents saturation, and for encouraging coarse coding and broad themes in order to achieve so-called data saturation rather than deep reflexive and interpretative engagement with the data(7).”

This includes an additional reference to the paper “To saturate or not to saturate? Questioning data saturation as an useful concept for thematic analysis and sample-size rationales” by Braun and Clarke.

3. *If detailed screening, eligibility, or refusal numbers were not collected, this should be acknowledged clearly as a limitation. The explanation that this was an interpretive qualitative study is reasonable, but a brief acknowledgement that the absence of these data limits assessment of recruitment flow and possible selection bias would strengthen transparency.*

Thank you for your recommendation. The following passage has been added at lines 657-663 to recognise although we would maintain as authors that our decision not to collect detailed screening and eligibility numbers is epistemologically and methodologically sound as the sampling approach was purposive rather than probability based, the absence of these data limits the assessment of the recruitment process and limits the readers’ ability to scrutinise for selection bias:

“During the recruitment process we did not collect detailed screening, eligibility, or refusal numbers. The reason for this is that our epistemiological stance for this work is interpretivists and that has informed our purposive sampling strategy. We aimed to recruit a rich sample that was representative of the range of experiences rather than a probability based statistically representative sample as would be sought in studies with a positivist approach. However, the absence of these data limits the assessment of recruitment flow or potential selection bias.”

4. *The expanded analysis section is much improved. The authors may consider adding one or two sentences clarifying how analytic disagreements or interpretive uncertainties were handled during coding clinics, and whether negative or divergent cases were actively considered when developing the final themes. This would further strengthen confidence in the analytic process.*

Thank you for your comment. The reality is that the authors who conducted the coding clinics did not diverge greatly in their application of the coding framework and the analytic disagreement was minor. This has been reflected in the addition of this following sentence at 132-134:

“Analytic disagreements between the two authors were rare, and were resolved through discussion.”

Perhaps unsurprisingly considering how narrow and defined our phenomenon of interest was, we found that although there was considerable heterogeneity in our population in terms of KRT modality, life circumstances, clinical progression, the severity and the evolution over time of their treatment burden there were no negative cases (i.e. participants that did not experience any burden) or cases that diverged greatly in the nature rather than the extent of their burden. As there were no cases where our themes did not fit, we did not have any negative or highly divergent cases to consider in the analysis. The following has been added at line 147-148 to reflect this:

“There were no negative or divergent cases that required active consideration during the analysis process.”

5. *The revised manuscript appropriately acknowledges heterogeneity across modality pathways. Please ensure that any remaining statements comparing transplant, haemodialysis, and peritoneal dialysis are phrased cautiously, given the small and clinically heterogeneous subgroups.*

Thank you for your recommendation. The following alterations have been made to phrase conclusions about subgroups more cautiously.

Line 558-560 now reads:

“Thirdly, successful transplantation can have a transformative effect on burden, offering the possibility of a much lower healthcare workload and increased capacity due to improved physical health.”

Line 693-695 now reads :

“Secondly, for eligible patients, successful kidney transplantation could represent the treatment pathway that offers the lowest workload and burden, and interventions that improve access to transplantation are key to reducing treatment burden”

*6. A final careful copyedit is recommended to address minor grammatical, formatting, and spacing issues. In the abstract, the phrase “Using a framework analysis informed by…” could be revised to “We used framework analysis informed by…” for grammatical clarity. Please also ensure consistent spelling and hyphenation of terms such as “KRT transition-related workload,” “healthcare workload,” and “healthcare system.”*

A final copyedit has been completed with several minor grammatical, formatting and spacing alterations.

Thank you for the opportunity to further improve our manuscript for consideration of publication. I look forward to your response.

Kind regards,

Dr Catrin H Jones
