# Supplementary material for: Exploration of treatment burden during transition onto kidney replacement therapy: A longitudinal qualitative study
Source: PLoS One. 2026 Oct 12;21(10):e0360290. doi: 10.1371/journal.pone.0360290 (PMC13657324; doi:10.1371/journal.pone.0360290)
Supplement: Peer review history

# Author comment: Author response to Decision Letter 0

**DOI:** 10.1371/journal.pone.0360290.r001

**Transfer Alert**

This paper was transferred from another journal. As a result, its full editorial history (including decision letters, peer reviews and author responses) may not be present.

30 Jan 2026

# Peer review: Decision Letter 0

**DOI:** 10.1371/journal.pone.0360290.r002

**Authors:** Wisit Kaewput

21 Mar 2026

Dear Dr. Jones,

Thank you for submitting your manuscript to PLOS ONE. After careful consideration, we feel that it has merit but does not fully meet PLOS ONE’s publication criteria as it currently stands. Therefore, we invite you to submit a revised version of the manuscript that addresses the points raised during the review process.

==============================

**ACADEMIC EDITOR:**

Major concerns include:

1. The manuscript concludes that “healthcare system processes are the main modifiable driver of treatment burden in this population.” While the data do support the importance of health system factors, this claim feels somewhat stronger than the evidence presented. Other important contributors are also evident in the data, including psychological resilience, family support, financial security, migration-related isolation, modality-related complications, and biographical disruption. The manuscript would be more convincing if it presented health system factors as a prominent and potentially modifiable contributor, rather than the single main driver.

2. The use of Normalisation Process Theory, Theory of Patient Capacity, and Burden of Treatment Theory is intellectually ambitious, but at times the analysis feels overconstructed. It is not always clear whether the themes emerged primarily from the data or were substantially shaped by the pre-existing frameworks. The manuscript moves across several overlapping constructs, workload, capacity, burden, agency, relational networks, mobilising capacity, delegated tasks, and system function, without always clearly distinguishing them. The authors should either simplify the conceptual framing or provide a clearer justification for why all three frameworks were necessary and what each uniquely adds.

3. Although the manuscript states that line-by-line coding was undertaken by the first author and that framework analysis was used, the analytic process remains somewhat insufficiently described. In particular, it would help readers to understand: how the initial coding framework was developed, the balance between deductive and inductive coding, how analytic decisions were discussed and refined, how information power was applied in determining sample sufficiency, how the five final themes were generated from the coded data, and how the longitudinal nature of the dataset shaped the final interpretation.

At present, the analysis reads as thoughtful and polished, but additional detail would improve its transparency and trustworthiness.

4. One of the notable strengths of the study is its repeated interviews over time, yet this aspect is not fully realized in the presentation of the findings. Although “evolution of burden over time” is identified as a cross-cutting theme, much of the Results section still reads in a largely cross-sectional way. More explicit illustration of how participants’ views, capacities, or burdens changed across timepoints would strengthen the manuscript considerably. The inclusion of more within-case longitudinal examples or trajectories would help demonstrate the added value of the study design.

5. The study is based in a single center in the West of Scotland, excludes people without sufficient English proficiency, and includes relatively small numbers within each modality subgroup. These limitations are acknowledged, but their implications for interpretation deserve greater emphasis. In particular, the exclusion of those requiring translation is important, as language barriers may themselves be a major source of treatment burden. Similarly, the limited ethnic diversity of the sample constrains the extent to which the findings can be generalized more broadly. The discussion should be somewhat more cautious in places where broader claims are made.

6. The manuscript contrasts HD, PD, and transplant experiences in several places, but the subgroups are small and clinically heterogeneous. Some participants changed modality, some underwent transplant after dialysis, and some were receiving dialysis as a bridge to transplantation. These are meaningful distinctions, and stronger acknowledgement of this heterogeneity is needed when interpreting modality-related differences. Statements suggesting that one pathway carries lower burden than another may be directionally reasonable, but should be presented more cautiously in light of the design and sample size.

7. The reflexivity statement is appreciated, but it is relatively brief. Given that the interviewer had a transplant/renal clinical background and prior knowledge of KRT, more reflection would be helpful on how this may have shaped data collection and interpretation. For example, this background may have influenced which issues were probed, how service-related concerns were interpreted, or how participants framed their experiences. A more developed reflexivity section would strengthen the credibility of the analysis.

8. The discussion includes broader recommendations around transplant access, continuity of care, system redesign, and capacity coaching. These are plausible and potentially important implications, but in some places the manuscript moves somewhat quickly from qualitative findings to broader policy or service recommendations. It would strengthen the paper to more clearly distinguish findings directly grounded in the data from broader implications or hypotheses for future research and service development.

Minor concerns include:

1. The manuscript would benefit from being reviewed against COREQ or a similar reporting checklist to ensure that all relevant details are fully reported.

2. Some ideas recur across the Results and Discussion, particularly around system accessibility, continuity, relational support, and evolving burden. Streamlining some sections may improve clarity and impact.

3. Phrases such as “main modifiable driver” and “best way to reduce burden” would benefit from more cautious phrasing.

4. There are minor editorial issues throughout. For example, “There are no competing interest” should be revised to “There are no competing interests,” and there are some formatting and spacing inconsistencies that should be corrected.

==============================

Please submit your revised manuscript by May 05 2026 11:59PM. If you will need more time than this to complete your revisions, please reply to this message or contact the journal office at plosone@plos.org. When you're ready to submit your revision, log on to https://www.editorialmanager.com/pone/ and select the 'Submissions Needing Revision' folder to locate your manuscript file.

- A letter that responds to each point raised by the academic editor and reviewer(s). You should upload this letter as a separate file labeled 'Response to Reviewers'.
- A marked-up copy of your manuscript that highlights changes made to the original version. You should upload this as a separate file labeled 'Revised Manuscript with Track Changes'.
- An unmarked version of your revised paper without tracked changes. You should upload this as a separate file labeled 'Manuscript'.

If you would like to make changes to your financial disclosure, please include your updated statement in your cover letter. Guidelines for resubmitting your figure files are available below the reviewer comments at the end of this letter.

If applicable, we recommend that you deposit your laboratory protocols in protocols.io to enhance the reproducibility of your results. Protocols.io assigns your protocol its own identifier (DOI) so that it can be cited independently in the future. For instructions see: https://journals.plos.org/plosone/s/submission-guidelines#loc-laboratory-protocols. Additionally, PLOS ONE offers an option for publishing peer-reviewed Lab Protocol articles, which describe protocols hosted on protocols.io. Read more information on sharing protocols at https://plos.org/protocols?utm_medium=editorial-email&utm_source=authorletters&utm_campaign=protocols.

We look forward to receiving your revised manuscript.

Kind regards,

Wisit Kaewput, MD, FRCP(London), FASN, FAcadMEd

Academic Editor

PLOS One

Journal Requirements:

When submitting your revision, we need you to address these additional requirements.

1. Please ensure that your manuscript meets PLOS ONE’s style requirements, including those for file naming. The PLOS ONE style templates can be found at

https://journals.plos.org/plosone/s/file?id=wjVg/PLOSOne_formatting_sample_main_body.pdf and

https://journals.plos.org/plosone/s/file?id=ba62/PLOSOne_formatting_sample_title_authors_affiliations.pdf

2. We note that the grant information you provided in the ‘Funding Information’ and ‘Financial Disclosure’ sections do not match.

When you resubmit, please ensure that you provide the correct grant numbers for the awards you received for your study in the ‘Funding Information’ section.

3. Thank you for stating the following financial disclosure:

CSO Clinical Academic Fellowship

At this time, please address the following queries:

a) Please clarify the sources of funding (financial or material support) for your study. List the grants or organizations that supported your study, including funding received from your institution.

b) State what role the funders took in the study. If the funders had no role in your study, please state: “The funders had no role in study design, data collection and analysis, decision to publish, or preparation of the manuscript.”

c) If any authors received a salary from any of your funders, please state which authors and which funders.

d) If you did not receive any funding for this study, please state: “The authors received no specific funding for this work.”

Please include your amended statements within your cover letter; we will change the online submission form on your behalf.

4. Please expand the acronym “CSO” (as indicated in your financial disclosure) so that it states the name of your funders in full.

This information should be included in your cover letter; we will change the online submission form on your behalf.

5. Thank you for stating the following in the Competing Interests section:

No competing interest

Please confirm that this does not alter your adherence to all PLOS ONE policies on sharing data and materials, by including the following statement: "This does not alter our adherence to PLOS ONE policies on sharing data and materials.” (as detailed online in our guide for authors http://journals.plos.org/plosone/s/competing-interests). If there are restrictions on sharing of data and/or materials, please state these. Please note that we cannot proceed with consideration of your article until this information has been declared.

Please include your updated Competing Interests statement in your cover letter; we will change the online submission form on your behalf.

6. We note that you have indicated that there are restrictions to data sharing for this study. For studies involving human research participant data or other sensitive data, we encourage authors to share de-identified or anonymized data. However, when data cannot be publicly shared for ethical reasons, we allow authors to make their data sets available upon request. For information on unacceptable data access restrictions, please see http://journals.plos.org/plosone/s/data-availability#loc-unacceptable-data-access-restrictions.

Before we proceed with your manuscript, please address the following prompts:

a) If there are ethical or legal restrictions on sharing a de-identified data set, please explain them in detail (e.g., data contain potentially identifying or sensitive patient information, data are owned by a third-party organization, etc.) and who has imposed them (e.g., a Research Ethics Committee or Institutional Review Board, etc.). Please also provide contact information for a data access committee, ethics committee, or other institutional body to which data requests may be sent.

b) If there are no restrictions, please upload the minimal anonymized data set necessary to replicate your study findings to a stable, public repository and provide us with the relevant URLs, DOIs, or accession numbers. Please see http://www.bmj.com/content/340/bmj.c181.long for guidelines on how to de-identify and prepare clinical data for publication. For a list of recommended repositories, please see https://journals.plos.org/plosone/s/recommended-repositories. You also have the option of uploading the data as Supporting Information files, but we would recommend depositing data directly to a data repository if possible.

Please update your Data Availability statement in the submission form accordingly.

7. Please amend your list of authors on the manuscript to ensure that each author is linked to an affiliation. Authors’ affiliations should reflect the institution where the work was done (if authors moved subsequently, you can also list the new affiliation stating “current affiliation:….” as necessary).

8. If the reviewer comments include a recommendation to cite specific previously published works, please review and evaluate these publications to determine whether they are relevant and should be cited. There is no requirement to cite these works unless the editor has indicated otherwise.

Additional Editor Comments:

This manuscript addresses an important and underexplored issue: treatment burden during transition to kidney replacement therapy (KRT). The longitudinal qualitative design, serial interviews, and engagement with relevant theoretical frameworks are all potentially valuable strengths. The topic is clinically meaningful, and the authors have clearly invested substantial effort in data collection and interpretation. However, in its current form, the manuscript has several conceptual, methodological, and reporting issues that limit confidence in the rigor of the analysis and the strength of some of the conclusions. I believe the study has promise, but substantial revision would be required before it is suitable for publication.

Major concerns include:

1. The manuscript concludes that “healthcare system processes are the main modifiable driver of treatment burden in this population.” While the data do support the importance of health system factors, this claim feels somewhat stronger than the evidence presented. Other important contributors are also evident in the data, including psychological resilience, family support, financial security, migration-related isolation, modality-related complications, and biographical disruption. The manuscript would be more convincing if it presented health system factors as a prominent and potentially modifiable contributor, rather than the single main driver.

2. The use of Normalisation Process Theory, Theory of Patient Capacity, and Burden of Treatment Theory is intellectually ambitious, but at times the analysis feels overconstructed. It is not always clear whether the themes emerged primarily from the data or were substantially shaped by the pre-existing frameworks. The manuscript moves across several overlapping constructs, workload, capacity, burden, agency, relational networks, mobilising capacity, delegated tasks, and system function, without always clearly distinguishing them. The authors should either simplify the conceptual framing or provide a clearer justification for why all three frameworks were necessary and what each uniquely adds.

3. Although the manuscript states that line-by-line coding was undertaken by the first author and that framework analysis was used, the analytic process remains somewhat insufficiently described. In particular, it would help readers to understand: how the initial coding framework was developed, the balance between deductive and inductive coding, how analytic decisions were discussed and refined, how information power was applied in determining sample sufficiency, how the five final themes were generated from the coded data, and how the longitudinal nature of the dataset shaped the final interpretation.

At present, the analysis reads as thoughtful and polished, but additional detail would improve its transparency and trustworthiness.

4. One of the notable strengths of the study is its repeated interviews over time, yet this aspect is not fully realized in the presentation of the findings. Although “evolution of burden over time” is identified as a cross-cutting theme, much of the Results section still reads in a largely cross-sectional way. More explicit illustration of how participants’ views, capacities, or burdens changed across timepoints would strengthen the manuscript considerably. The inclusion of more within-case longitudinal examples or trajectories would help demonstrate the added value of the study design.

5. The study is based in a single center in the West of Scotland, excludes people without sufficient English proficiency, and includes relatively small numbers within each modality subgroup. These limitations are acknowledged, but their implications for interpretation deserve greater emphasis. In particular, the exclusion of those requiring translation is important, as language barriers may themselves be a major source of treatment burden. Similarly, the limited ethnic diversity of the sample constrains the extent to which the findings can be generalized more broadly. The discussion should be somewhat more cautious in places where broader claims are made.

6. The manuscript contrasts HD, PD, and transplant experiences in several places, but the subgroups are small and clinically heterogeneous. Some participants changed modality, some underwent transplant after dialysis, and some were receiving dialysis as a bridge to transplantation. These are meaningful distinctions, and stronger acknowledgement of this heterogeneity is needed when interpreting modality-related differences. Statements suggesting that one pathway carries lower burden than another may be directionally reasonable, but should be presented more cautiously in light of the design and sample size.

7. The reflexivity statement is appreciated, but it is relatively brief. Given that the interviewer had a transplant/renal clinical background and prior knowledge of KRT, more reflection would be helpful on how this may have shaped data collection and interpretation. For example, this background may have influenced which issues were probed, how service-related concerns were interpreted, or how participants framed their experiences. A more developed reflexivity section would strengthen the credibility of the analysis.

8. The discussion includes broader recommendations around transplant access, continuity of care, system redesign, and capacity coaching. These are plausible and potentially important implications, but in some places the manuscript moves somewhat quickly from qualitative findings to broader policy or service recommendations. It would strengthen the paper to more clearly distinguish findings directly grounded in the data from broader implications or hypotheses for future research and service development.

Minor concerns include:

1. The manuscript would benefit from being reviewed against COREQ or a similar reporting checklist to ensure that all relevant details are fully reported.

2. Some ideas recur across the Results and Discussion, particularly around system accessibility, continuity, relational support, and evolving burden. Streamlining some sections may improve clarity and impact.

3. Phrases such as “main modifiable driver” and “best way to reduce burden” would benefit from more cautious phrasing.

4. There are minor editorial issues throughout. For example, “There are no competing interest” should be revised to “There are no competing interests,” and there are some formatting and spacing inconsistencies that should be corrected.

[Note: HTML markup is below. Please do not edit.]

Reviewers' comments:

Reviewer’s Responses to Questions

**Comments to the Author**

1. Is the manuscript technically sound, and do the data support the conclusions?

Reviewer #1: Yes

Reviewer #2: Partly

Reviewer #3: Yes

**********

2. Has the statistical analysis been performed appropriately and rigorously?

Reviewer #1: Yes

Reviewer #2: N/A

Reviewer #3: Yes

**********

3. Have the authors made all data underlying the findings in their manuscript fully available?

The PLOS Data policy

Reviewer #1: No

Reviewer #2: Yes

Reviewer #3: Yes

**********

4. Is the manuscript presented in an intelligible fashion and written in standard English?

Reviewer #1: Yes

Reviewer #2: No

Reviewer #3: Yes

**********

Reviewer #1: this is a strong qualitative manuscript that makes a meaningful contribution. I would support publication after minor revisions focused on clarifying a few methodological details, tightening the Data Availability Statement, and addressing small language/formatting issues.

Reviewer #2: This is a thoughtful and clinically relevant qualitative study with strong theoretical grounding and meaningful implications for patient-centered kidney care. With greater methodological transparency and more explicit system-level recommendations, the manuscript would make a significant contribution to understanding and reducing treatment burden during KRT transition.

Minor Comments:

-Clarify whether participants were pre-dialysis, initiating dialysis, undergoing transplantation, or experiencing modality changes, as these contexts may differ significantly in burden.

-Define “treatment burden” explicitly at first mention for readers unfamiliar with the concept.

-Provide more detail about reflexivity—how researchers’ backgrounds may have influenced data collection and interpretation.

-Include information on ethical approval and consent procedures.

-Consider adding a conceptual diagram summarizing the interaction between workload, capacity, and system factors.

-Minor language refinement is recommended to improve clarity and reduce repetition in the Results section.

Major Comments

-Originality and Contribution

The study addresses an important and relatively underexplored aspect of kidney replacement therapy (KRT) transition by examining it through the framework of treatment burden. The conceptual framing is strong; however, the manuscript would benefit from a clearer articulation of how this work advances existing literature. Please specify what is novel about examining KRT transition longitudinally and how this differs from prior qualitative studies in nephrology populations.

-Sampling and Participant Characteristics

Greater detail is needed regarding participant demographics and clinical characteristics (e.g., age, sex, socioeconomic background, type of KRT initiated, transplant vs dialysis pathway). Clarify the sampling strategy (purposive? maximum variation?) and how diversity of experiences was ensured. Additionally, discuss whether thematic saturation was achieved and how this was determined.

-Use of Theoretical Frameworks

The integration of Normalisation Process Theory, Theory of Patient Capacity, and Burden of Treatment Theory is a strength, but the operationalization of these frameworks requires further clarification. How were these theories applied during coding and analysis? Were they used deductively, inductively, or in combination? Providing a clearer analytic pathway would enhance methodological transparency.

-Longitudinal Design and Evolution of Burden

The serial interview design is valuable; however, the manuscript should more explicitly demonstrate how treatment burden evolved over time. Consider including illustrative trajectories or case examples that show change across interviews. Clarify the time intervals between interviews and how transition phases were defined.

-Healthcare System as a Modifiable Driver

The conclusion identifies healthcare system processes as the main modifiable driver of treatment burden. While this is compelling, more specific examples are needed. What particular system factors (e.g., appointment scheduling, communication gaps, education provision) were most burdensome? Concrete recommendations for practice or service redesign would strengthen translational impact.

Reviewer #3: Dear authors

Thank you for this well-conducted, important study providing deep insight into what shapes the burden of KRT on patients, addressing an important topic for ensuring the best quality of care for patients undergoing KRT. Below are some recommended modifications and comments for your consideration;

1- Define abbreviations in tables and text once before using; (HD, PD and KTx, PDM and KTF, KDOQI, etc..)

2- Purposive stratified sampling is claimed, but no information on: How many patients were screened/approached? How many were eligible? How many declined and why? Final recruitment rate per stratum?

3- The study has a longitudinal design; however, results are displayed as summaries of the entire period. More clarifications on what each interview produced, and how prevalent each theme was during each period, as well as the progression of patients’ comments and burden perceptions wherever possible could prove beneficial.

4- While the study includes interviews up to 12 months post-initiation, much of the themes analyzed are focusing on the transitional period, further clarification of how post-initiation experiences differed from early transition could strengthen the longitudinal design of the paper.

5- Inadequate description of analytic trustworthiness. No mention of: Independent double-coding of any transcripts Inter-coder agreement/reliability procedures Audit trail Deviant case analysis Member checking.

6- Kappa must be reported for above point.

7- Discussion can be toned down, strong conclusions, healthcare system processes are the main modifiable driver need explicit transferability discussion. This is a single NHS center study.

8- Despite stratification, very low non-White representation. Must be addressed as limitation.

**********

what does this mean?). If published, this will include your full peer review and any attached files.

If you choose “no”, your identity will remain anonymous but your review may still be made public.

**Do you want your identity to be public for this peer review?**  For information about this choice, including consent withdrawal, please see our Privacy Policy

Reviewer #1: **Yes:**  Malak Abdulqadir

Reviewer #2: **Yes:**  ahmed alshewered

Reviewer #3: No

**********

[NOTE: If reviewer comments were submitted as an attachment file, they will be attached to this email and accessible via the submission site. Please log into your account, locate the manuscript record, and check for the action link "View Attachments". If this link does not appear, there are no attachment files.]

To ensure your figures meet our technical requirements, please review our figure guidelines: https://journals.plos.org/plosone/s/figures

You may also use PLOS’s free figure tool, NAAS, to help you prepare publication quality figures: https://journals.plos.org/plosone/s/figures#loc-tools-for-figure-preparation.

NAAS will assess whether your figures meet our technical requirements by comparing each figure against our figure specifications.

# Author comment: Author response to Decision Letter 1

**DOI:** 10.1371/journal.pone.0360290.r003

12 May 2026

School of Health and Wellbeing

Clarice Pears Building

90 Byers Road

Glasgow

03/05/2026

Dear Editor,

RE: Exploration of treatment burden during transition onto kidney replacement therapy: a longitudinal qualitative study. PONE-D-26-04817

Thank you for your detailed and thoughtful feedback on our manuscript, and for the opportunity to revise our work for further consideration. We have made the following revisions to the work in response to the points raised by the academic editor.

1. The manuscript concludes that “healthcare system processes are the main modifiable driver of treatment burden in this population.” While the data do support the importance of health system factors, this claim feels somewhat stronger than the evidence presented. Other important contributors are also evident in the data, including psychological resilience, family support, financial security, migration-related isolation, modality-related complications, and biographical disruption. The manuscript would be more convincing if it presented health system factors as a prominent and potentially modifiable contributor, rather than the single main driver.

This is an extremely helpful comment. It was our intention to argue that health system processes are important for health professionals and policy makers to consider because they are within their sphere of influence when other factors such as family support may not be, rather than being the only driver treatment burden and we have made this clearer.

The following modifications have been made to the manuscript:

Conclusion in the abstract (pg 2, line 29-31) has been amended from “Findings support that healthcare system processes are the main modifiable driver of treatment burden in this population and clinicians should strive to develop treatment burden informed care for KRT transition” to “Transitioning onto KRT is a process that entails a high volume of healthcare workload and significant treatment burden. Experiences of treatment burden are influenced by the nature of the work required; the patient’s capacity including physical, psychological, financial and cognitive factors; the capacity of their social network; and the functioning of the healthcare system. Particular focus should be placed on understanding the impact of the healthcare system on experiences of burden, as it represents a potentially modifiable driver of treatment burden that clinicians and healthcare policy makers can directly influence.”

Page 35 line 596-600 has been changed from: “Understanding the impact of health system function on burden is key it is a potentially modifiable factor that clinicians and policy makers can directly impact whereas other factors that are the product of the wider socioeconomic functioning of society are more intractable.” to “Whilst treatment burden is multifactorial and influenced by many factors relating to the patient, their social network, and the healthcare system, understanding the impact of health system function on burden is important as it is a potentially modifiable factor that clinicians and policy makers can directly impact whereas other factors that are the product of the wider socioeconomic functioning of society are more intractable.”

Page 38 line 652-654 has been changed from “A key finding is that healthcare system processes are the main modifiable driver of treatment burden in this population and clinicians should strive to develop treatment burden informed care for KRT transition” to “A key finding is that healthcare system processes are a prominent contributor to the experience of burden, and represent a potentially modifiable driver of treatment burden in this population. Developing treatment burden informed care for KRT transition could represent a potent way to reduce the burden experienced by patients.”

2. The use of Normalisation Process Theory, Theory of Patient Capacity, and Burden of Treatment Theory is intellectually ambitious, but at times the analysis feels overconstructed. It is not always clear whether the themes emerged primarily from the data or were substantially shaped by the pre-existing frameworks. The manuscript moves across several overlapping constructs, workload, capacity, burden, agency, relational networks, mobilising capacity, delegated tasks, and system function, without always clearly distinguishing them. The authors should either simplify the conceptual framing or provide a clearer justification for why all three frameworks were necessary and what each uniquely adds.

Many thanks for this insightful comment. We recognise that this is a theory- heavy paper, but we do feel that the three theories add to the work. Firstly, it is important to emphasise that these theories are interrelated and there are overlapping constructs between NPT and BoTT. The description of the conceptualisation process is an accurate reflection of how we sought to understand the data: we started with NPT and TPC to understand workload and capacity as these theories facilitated a detailed exploration of those concepts and the factors that influence them. For this we used a framework analysis technique, utilising a framework underpinned by NPT and TPC with careful attention paid to any data that fell outside these frameworks. Data related to evolution of burden over time were noted as important and therefore added to the analysis. The subsequent use of BoTT for a higher order analysis allowed exploration of the relationships between healthcare workload, capacity and the healthcare system, which we felt was important to assess. In a systematic review that was a precursor to this work we only used NPT and TPC, and developed a taxonomy of workload and a taxonomy of capacity that informed the topic guides used for this work(1). However when we applied this to the longitudinal data although there was rich data on workload and capacity and the framework fitted well, we felt that how the interaction between different components of workload and capacity created treatment burden and how this evolved over time was best understood within the framework of BoTT. Please note that our team have a deep understanding and experience of using these theories, which facilitated the complex analysis. Dr Gallacher is considered an expert in the use of these theories and in the field of treatment burden: she was co-author on the paper that describes the development of BoTT(2); has used NPT extensively in her research(3–7); and has co-authored a paper that describes the development, context, and relationship of the theories used in this work(8). Advice was also sought on data analysis from Prof Carl May who led on the development of both NPT and BoTT, this has now been added as an acknowledgement in the paper.

3. Although the manuscript states that line-by-line coding was undertaken by the first author and that framework analysis was used, the analytic process remains somewhat insufficiently described. In particular, it would help readers to understand: how the initial coding framework was developed, the balance between deductive and inductive coding, how analytic decisions were discussed and refined, how information power was applied in determining sample sufficiency, how the five final themes were generated from the coded data, and how the longitudinal nature of the dataset shaped the final interpretation.

At present, the analysis reads as thoughtful and polished, but additional detail would improve its transparency and trustworthiness.

Thank you for these comments. The initial coding framework was developed using a published NPT coding manual (9) and a prior systematic review(1) we had conducted that utilised NPT and TPC to examine healthcare workload and capacity in the same patient population as this manuscript. These frameworks were applied deductively, but with inductive coding of any concepts that fell outwith the initial coding frame. The manuscript has been amended with the addition of this line to further clarify how the initial coding frame was developed (pg 7 line 124-128):

“The initial coding framework was developed informed by a published NPT coding manual and a prior systematic review and applied deductively. Any concept falling outwith the NPT and TPC were coded separately, and specific separate codes were developed iteratively to characterise changes to workload, capacity and burden over time.”

The balance between deductive and inductive coding has been further clarified with the addition of this line (page 7 line 128-130): “There were no workload or capacity concepts that fell outwith the initial coding frame, but all transcripts had themes relating to the evolution of burden over time which were coded inductively.”

We have expanded on how we used information power to determine sample size with the addition of this paragraph (page 5, line 91-101): “Information power was used to guide our sample size(10). Five factors were considered: study aim, sample specificity, use of theory, quality of dialogue, and analysis strategy. We also considered the risk of clinical deterioration or death of participants during the study period due to the nature of advanced kidney disease. The study has a narrow and focused aim, and although we needed to capture a range of modalities the population of interest was highly specific. The longitudinal design with rich, in depth interviews over time gave us confidence that the quality of the dialogue was likely to be high, and the framework approach grounded in previous work gave us confidence that the analysis strategy was likely to have a high yield. We therefore estimated a sample size between 25-40 followed up for 12 months would provide sufficient information power whilst remaining feasible, and we stopped recruiting at 30 participants.”

We have now clarified how our themes came to be developed. The first four themes are directly informed by BoTT- they are the four domains applied in the context of KRT transition. The fifth theme directly comes from the coding of how burden evolved over time. The manuscript has been altered from : “We subsequently applied the BoTT domains of mobilising capacity, expressing capacity, mobilizing for delegated tasks and enacting delegated tasks to these taxonomies to develop a higher order analysis of how workload and capacity interact in the context of a healthcare system and to describe the evolution of treatment burden during KRT transition” to “We subsequently applied the BoTT domains of mobilising capacity, expressing capacity, mobilizing for delegated tasks and enacting delegated tasks to the coding framework along with an additional higher order theme that pertained to the evolution of burden over time. This higher order analysis of how workload and capacity interact in the context of a healthcare system and the evolution of treatment burden over time during KRT transition informed our five themes.

4. One of the notable strengths of the study is its repeated interviews over time, yet this aspect is not fully realized in the presentation of the findings. Although “evolution of burden over time” is identified as a cross-cutting theme, much of the Results section still reads in a largely cross-sectional way. More explicit illustration of how participants’ views, capacities, or burdens changed across timepoints would strengthen the manuscript considerably. The inclusion of more within-case longitudinal examples or trajectories would help demonstrate the added value of the study design.

Thank you for your helpful comment. We have made the following alterations:

We have introduced a new figure, figure 6, to illustrate different trajectories in the evolution of treatment burden over time, and Table 4 which has with three illustrative vignettes/case studies to better illustrate the evolution of burden over time. The first sentences have been amended to be phrased more cautiously regarding the impact of the healthcare system and to introduce Table 4 (page 25, line 452-455):

“When evaluating the evolution of burden over time in longitudinal interviews, the interaction between individual, social network, and healthcare system factors and the progress and outcomes of the healthcare work undertaken heavily influenced ongoing burden (figure 6). We have further illustrated this with three clinical vignettes (Table 4).”

5. The study is based in a single center in the West of Scotland, excludes people without sufficient English proficiency, and includes relatively small numbers within each modality subgroup. These limitations are acknowledged, but their implications for interpretation deserve greater emphasis. In particular, the exclusion of those requiring translation is important, as language barriers may themselves be a major source of treatment burden. Similarly, the limited ethnic diversity of the sample constrains the extent to which the findings can be generalized more broadly. The discussion should be somewhat more cautious in places where broader claims are made.

Thank you. Discussion of the impact of not being able to provide translation services has been expanded to now read (Page 36, line 620-626):

“We were unable to offer translation services due to financial constraints and therefore were only able to recruit those whose English was sufficient to conduct interviews without support. This is a significant limitation as it does not capture the treatment burden generated by navigating a healthcare service with limited proficiency in the dominant local language. Language barriers could represent a major direct and indirect source of treatment burden, and this has not been addressed by this work.”

The limited ethnic diversity of the participants has been more explicitly discussed to both acknowledge it’s potential limitation but also to emphasise that the study population is representative of the ethnicity proportions of the unit patient population. At line 626 the following has been added:

“The ethnic diversity in our study population (90% White, 10% Asian) is representative of the ethnic diversity of the wider patient population served by the unit, but is not representative of the ethnic diversity of the renal population in the UK more widely which again limits the generalisability of the study.”

6. The manuscript contrasts HD, PD, and transplant experiences in several places, but the subgroups are small and clinically heterogeneous. Some participants changed modality, some underwent transplant after dialysis, and some were receiving dialysis as a bridge to transplantation. These are meaningful distinctions, and stronger acknowledgement of this heterogeneity is needed when interpreting modality-related differences. Statements suggesting that one pathway carries lower burden than another may be directionally reasonable, but should be presented more cautiously in light of the design and sample size.

Thank you for your comment. The clinical pathways within the study sample are representative of the clinical pathways experienced by patients transitioning onto KRT in developed countries with access to both dialysis and transplantation(11). The heterogeneity of these widely recognisable clinical pathways within this study cohort expands the primary data in the literature in the exploration and understanding of the treatment burden experienced by patients transitioning onto KRT. We feel that the argument that improving access to transplant is a vital way of reducing burden is both an important one and one that is grounded within our data, and one that we are keen to retain within the paper. Although our conceptualisation of the patient experience in KRT as treatment burden is new, our findings that transplantation is associated with notably superior outcomes for patients is in keeping with previous research regarding the benefits of transplant. We have modified our manuscript to present our finding more cautiously, and to emphasise that our conclusion has been reached both from our data and in the context of other work, and therefore a higher degree of confidence can be placed in its validity.

Line 579 has been changed from “Our study has a clear signal that improving the access to transplantation for those who are potentially eligible is the best way to reduce burden at the level of the individual.” To “Our findings suggest that improving the access to transplantation for those who are potentially eligible may represent the most potent intervention to reduce burden at the level of the individual. This adds to the body of evidence that kidney transplantation is associated with both an improvement in quality of life from pre-transplant and a superior quality of life compared to HD or PD, and that seeking to achieve early transplantation offers the best outcomes for suitable patients.”

At line 630 the heterogeneity of the population has been discussed with the addition of:

“The heterogeneity of the study population represents both a strength and a weakness. The study population represents different modalities and 8 out of 30 participants experience a modality change. This could be seen as a strength as it reflects the real life experiences of KRT transition: patients rarely fit into neat fixed categories but experience complications, delays, successful transplantations, and many other changes to their clinical and life circumstance and this range of experiences are reflected in the data. However, the heterogeneity of the population has meant that some subgroups are small and therefore caution should be exercised in drawing too definitive a conclusion from the findings.”

7. The reflexivity statement is appreciated, but it is relatively brief. Given that the interviewer had a transplant/renal clinical background and prior knowledge of KRT, more reflection would be helpful on how this may have shaped data collection and interpretation. For example, this background may have influenced which issues were probed, how service-related concerns were interpreted, or how participants framed their experiences. A more developed reflexivity section would strengthen the credibility of the analysis.

Thank you for your comment. The reflexivity statement was kept brief due to concerns around the length of the paper. It has now been expanded to include this passage (page 8, line 147-160) : “Having a clinical background may have had both a positive and negative effect on the interviews and the analysis. Familiarity with kidney disease and its treatment could mean that patients felt at ease discussing complexities and nuances in their treatment journey confident that they would be understood. It could also aid with the analysis due to a prior deep understanding of the subject matter, and mean that the researcher is less likely to misunderstand or misinterpret the participants clinical course than a researcher without a relevant clinical background. However, it is possible that having a clinical background in KRT care could have negatively influenced the interviews and their analysis. Participants may have been more guarded in criticising the service or the staff. When analysing the data, it is possible that independent professional relationships with the clinicians involved and prior understanding of the strength and weaknesses of the KRT services from previously working in the unit may have sub-consciously coloured the interpretation of the data.”

8. The discussion includes broader recommendations around transplant access, continuity of care, system redesign, and capacity coaching. These are plausible and potentially important implications, but in some places the manuscript moves somewhat quickly from qualitative findings to broader policy or service recommendations. It would strengthen the paper to more clearly distinguish findings directly grounded in the data from broader implications or hypotheses for future research and service development.

Thank you for your comment. We have clarified our discussion so that it has three sections: ‘Summary of findings’, which is grounded in our direct findings; ‘How it fits with other work’, where we place our findings in the context of other published works; and ‘Implications for clinical practice, policy, and further research’ where broader policy and practice implications are discussed.

Minor concerns include:

1. The manuscript would benefit from being reviewed against COREQ or a similar reporting checklist to ensure that all relevant details are fully reported.

Thank you. We did use COREQ in the writing of our paper and should have provided the checklist as supplementary material, which we have now done.

2. Some ideas recur across the Results and Discussion, particularly around system accessibility, continuity, relational support, and evolving burden. Streamlining some sections may improve clarity and impact.

Thank you for your comment. We have made amendments in line with point 8 in the major concerns that hopefully addresses this.

3. Phrases such as “main modifiable driver” and “best way to reduce burden” would benefit from more cautious phrasing.

Thank you, these have been amended throughout with more guarded phrasing.

4. There are minor editorial issues throughout. For example, “There are no competing interest” should be revised to “There are no competing interests,” and there are some formatting and spacing inconsistencies that should be corrected.

Thank you. These have been amended.

We would like to extend our thanks to the three reviewers for their thoughtful comments. Many of the comments overlap with each other and with the recommendations of the academic reviewer therefore for brevity where issues have been previously addressed we have not duplicated our response. Reviewer 1 did not make any specific recommendations that had not been addressed by the response to the academic editor.

Reviewer 2 comments:

Minor Comments:

1)Clarify whether participants were pre-dialysis, initiating dialysis, undergoing transplantation, or experiencing modality changes, as these contexts may differ significantly in burden.

Thank you for your comment. We have provided a further figure (figure 1) illustrating the changes between modalities seen in our population, and provided a further figure (figure 6) and illustrative clinical vignettes (table 4) for further illustrate how different clinical trajectories may impact burden.

2)Define “treatment burden” explicitly at first mention for readers unfamiliar with the concept.

Thank you. We have already provided the following definition of treatment burden in the second sentence of the introduction: “Treatment burden is the impact of healthcare workload on patient wellbeing” (Line 38).

3)Provide more detail about reflexivity—how researchers’ backgrounds may have influenced data collection and interpretation.

Thank you- this has been addressed in Major Concern 7 above and the manuscript has been amended to provide further reflection of the impact of the researchers’ background on data collection and analysis.

4) Include information on ethical approval and consent procedures.

Thank you. Line 78-80 already detail the ethical approval and consent procedure and reads:

“Ethical approval was granted by the Proportionate Review Sub-Committee of the North West Preston Research Ethics Committee, IRAS project ID 322865, REC reference 23/NW/0267. Written consent was obtained from all participants.”

5) Consider adding a conceptual diagram summarizing the interaction between workload, capacity, and system factors.

Thank you. We have now included an additional figure, figure 6, to address this.

6)Minor language refinement is recommended to improve clarity and reduce repetition in the Results section.

Thank you, this has been addressed in response to the academic editor’s recommendations.

Major Comments

1)Originality and Contribution

The study addresses an important and relatively underexplored aspect of kidney replacement therapy (KRT) transition by examining it through the framework of treatment burden. The conceptual framing is strong; however, the manuscript would benefit from a clearer articulation of how this work advances existing literature. Please specify what is novel about examining KRT transition longitudinally and how this differs from prior qualitative studies in nephrology populations.

Thank you for your comment. We have added a paragraph in the discussion acknowledging that the longitudinal nature of our study is novel and stating our findings (page 32, line 517-533):

“The study of the evolution of burden over time in this work is novel and has not been explored in other work. We identified three main treatment burden patterns over time (figure 6). Firstly, the general trend is for healthcare workload and treatment burden to increase rapidly during the period immediately preceding KRT initiation, peak at the time of initiation, and subsequently reduce as the workload becomes normalised and the burden subsides. The nature of the workload also evolves, transitioning from initially sense-making and preparatory co-ordinating work before starting, to more task based enacting and co-ordinating work at the time of initiation, and eventually to more refining and reflexive work when established on KRT. Secondly, for some patients, treatment burden remains high and they do not experience the transition to greater stability and lower burden. This is predominantly driven by instability through complications, modality changes, and poor systemic processes. This instability means that participants could not progress to the refining and reflecting stage as they were perpetually required to understand and make decisions about new treatment avenues and co-ordinate their social network to enact new tasks in response to events, leading to sustained high burden. Thirdly, successful transplantation can have a transformative effect on burden, with a much lower healthcare workload and increased capacity due to improved physical health.”

2)Sampling and Participant Characteristics

Greater detail is needed regarding participant demographics and clinical characteristics (e.g., age, sex, socioeconomic background, type of KRT initiated, transplant vs dialysis pathway). Clarify the sampling strategy (purposive? maximum variation?) and how diversity of experiences was ensured. Additionally, discuss whether thematic saturation was achieved and how this was determined.

Thank you for your comment. We have already provided a detailed breakdown of the participant demographics (table 2), and each quote is identifiable by modality (HD/PD/KT) and gender (M/F). We are hesitant to provide more granular information such as a description of age, gender, ethnicity and starting modality of each participant in a table as this is a study from a single identifiable centre during a defined time period and thus providing more granular information could make participants identifiable and breach the terms of our ethical approval.

The sampling strategy had already been described at line 85-87:

“Participants were selected using a purposive stratified sampling approach; stratified by age, gender, starting modality, planned or unplanned start, and ethnicity.”

We have provided an additional sentence to clarify the aim of our strategy (line 87):

“The aim of the sampling strategy was to capture a diverse range of experiences of KRT transition.”

We have addressed information power in response to the academic editor’s recommendations.

3)Use of Theoretical Frameworks

The integration of Normalisation Process Theory, Theory of Patient Capacity, and Burden of Treatment Theory is a strength, but the operationalization of these frameworks requires further clarification. How were these theories applied during coding and analysis? Were they used deductively, inductively, or in combination? Providing a clearer analytic pathway would enhance methodological transparency.

Thank you. This has been addressed in response to the academic editor’s recommendations.

4)Longitudinal Design and Evolution of Burden

The serial interview design is valuable; however, the manuscript should more explicitly demonstrate how treatment burden evolved over time. Consider including illustrative trajectories or case examples that show change across interviews. Clarify the time intervals between interviews and how transition phases were defined.

Thank you. This has been addressed in response to the academic editor’s recommendations.

5)Healthcare System as a Modifiable Driver

The conclusion identifies healthcare system processes as the main modifiable driver of treatment burden. While this is compelling, more specific examples are needed. What particular system factors (e.g., appointment scheduling, communication gaps, education provision) were most burdensome? Concrete recommendations for practice or service redesign would strengthen translational impact.

Thank you for your comment. In our discussion we have identified three areas where poor system functioning can increase treatment burden (lack of continuity of care, poor interface between different system components, divergence between expectations and experience). In the section titled ‘Implications for clinical practice, policy, and further research’ we have identified improving access to transplant, improving continuity of care, ensuring seamless communication between different system components, and building capacity for the system to be more flexible around the needs of the patient as key areas for targeted intervention. In line with the reservations expressed by other reviewers of overstating conclusions regarding health system functioning from a one centre study, we are hesitant to state further concrete recommendations.

Reviewer 3 comments:

1- Define abbreviations in tables and text once before using; (HD, PD and KTx, PDM and KTF, KDOQI, etc..)

Thank you. This has been done and a list of abbreviations has been provided at the end.

2- Purposive stratified sampling is claimed, but no information on: How many patients were screened/approached? How many were eligible? How many declined and why? Final recruitment rate per stratum?

Thank you for this comment. Our epistemiological stance for this work is interpretivists and that has informed our purposive sampling strategy, aiming to recruit a rich sample that is representative of the range of experiences rather than a probability based statistically representative sample as would be sought in studies with a positivist approach. In keeping with thus epistemiologocal stance we have not collected information in number of patients screened/ approached/ eligible, reasons for declining or recruitment rate.

3- The study has a longitudinal design; however, results are displayed as summaries of the entire period. More clarifications on what each interview produced, and how prevalent each theme was during each period, as well as the progression of patients’ comments and burden perceptions wherever possible could prove beneficial.

Thank you- we have expanded on theme 5 which is the evolution of burden over time. This is discussed in more detail in the response to the academic editor.

4- While the study includes interviews up to 12 months post-initiation, much of the themes analyzed are focusing on the transitional period, further clarification of how post-initiation experiences differed from early transition could strengthen the longitudinal design of the paper.

Thank you for your comment. The evolution of burden over time has been further expanded in theme 5 as described in response to the academic editor.

5- Inadequate description of analytic trustworthiness. No mention of: Independent double-coding of any transcripts Inter-coder agreement/reliability procedures Audit trail Deviant case analysis Member checking.

Thank you. We have not reported on these as this was not the epistemological approach we chose to undertake (these are quality indicators rooted in a positivist epistemological stance). Transcripts were not double coded independently, but as stated in line 123-125 regular coding clinics was held between the first author (CJ) and senior authors (KG and KS) where coding was reviewed and emerging themes discussed in an iterative process. We would align with the arguments made by Braun and Clarke, who reject the concept of ‘coding reliability’ that relies on independent coders as an indication of trustworthiness. They argue that coding should be an interpretive, evolving, and collaborative process where findings arise from deep engagement with the data(12).

6- Kappa must be reported for above point.

Thank you. We did not calculate Kappa as the coding and theme development was conducted as an iterative process with discussion between the researchers therefore it would be inappropriate to calculate inter-coder agreement.

7- Discussion can be toned down, strong conclusions, healthcare system processes are the main modifiable driver need explicit transferability discussion. This is a single NHS center study.

Thank you. This has been addressed in response to the academic editor feedback and the language has been moderated throughout.

8- Despite stratification, very low non-White representation. Must be addressed as limitation.

Thank you for raising this important concern. We have amended the discussion of the limitations of this study in order to explicitly address the low ethnic diversity in our patient population and acknowledge the impact of this on the generalisability of our work (lines 619-629):

“Firstly, it is a single centre study conducted in the West of Scotland which limits its generalisability to other cultural contexts. We were unable to offer translation services due to financial constraints and therefore were only able to recruit those whose English was sufficient to conduct interviews without support. This is a significant limitation as it does not capture the treatment burden generated by navigating a healthcare service with limited proficiency in the dominant local language. Language barriers could represent a major direct and indirect source of treatment burden, and this has not been addressed by this work. The ethnic diversity in the study population (90% White, 10% Asian) is representative of the ethnic diversity of the wider patient population served by the unit, but is not representative of the ethnic diversity of the renal population in the UK more widely which again limits the generalisability of the study.”

We have made the following amendments in line with the journal requirements:

1. Please ensure that your manuscript meets PLOS ONE’s style requirements, including those for file naming.

Thank you-the necessary amendments have been made.

2. We note that the grant information you provided in the ‘Funding Information’ and ‘Financial Disclosure’ sections do not match.

Thank you- the cover letter has now been amended.

3. Thank you for stating the following financial disclosure:

CSO Clinical Academic Fellowship

At this time, please address the following queries:

a) Please clarify the sources of funding (financial or material support) for your study. List the grants or organizations that supported your study, including funding received from your institution.

b) State what role the funders took in the study. If the funders had no role in your study, please state: “The funders had no role in study design, data collection and analysis, decision to publish, or preparation of the manuscript.”

c) If any authors received a salary from any of your funders, please state which authors and which funders.

d) If you did not receive any funding for this study, please state: “The authors received no specific funding for this work.”

Please include your amended statements within your cover letter; we will change the online submission form on your behalf.

Thank you- the manuscript and cover letter has now been amended.

4. Please expand the acronym “CSO” (as indicated in your financial disclosure) so that it states the name of your funders in full.

This information should be included in your cover letter; we will change the online submission form on your behalf.

Thank you- the manuscript and cover letter has now been amended.

5. Thank you for stating the following in the Competing Interests section:

No competing interest

Please confirm that this does not alter your adherence to all PLOS ONE policies on sharing data and materials, by including the following statement: "This does not alter our adherence to PLOS ONE policies on sharing data and materials.” (as detailed online in our guide for authors http://journals.plos.org/plosone/s/competing-interests). If there are restrictions on sharing of data and/or materials, please state these. Please note that we cannot proceed with consideration of your article until this information has been declared.

Please include your updated Competing Interests statement in your cover letter; we will change the online submission form on your behalf.

Thank you, this has been amended.

6. We note that you have indicated that there are restrictions to data sharing for this study. For studies involving human research participant data or other sensitive data, we encourage authors to share de-identified or anonymized data. However, when data cannot be publicly shared for ethical reasons, we allow authors to make their data sets available upon request. For information on unacceptable data access restrictions, please see http://journals.plos.org/plosone/s/data-availability#loc-unacceptable-data-access-restrictions.

Before we proceed with your manuscript, please address the following prompts:

a) If there are ethical or legal restrictions on sharing a de-identified data set, please explain them in detail (e.g., data contain potentially identifying or sensitive patient information, data are owned by a third-party organization, etc.) and who has imposed them (e.g., a Research Ethics Committee or Institutional Review Board, etc.). Please also provide contact information for a data access committee, ethics committee, or other institutional body to which data requests may be sent.

b) If there are no restrictions, please upload the minimal anonymized data set necessary to replicate your study findings to a stable, public repository and provide us with the relevant URLs, DOIs, or accession numbers. Please see http://www.bmj.com/content/340/bmj.c181.longfor guidelines on how to de-identify and prepare clinical data for publication. For a list of recommended repositories, please see https://journals.plos.org/plosone/s/recommended-repositories. You also have the option of uploading the data as Supporting Information files, but we would recommend depositing data directly to a data repository if possible.

Please update your Data Availability statement in the submission form accordingly.

The terms of our ethical approval include secondary analysis of the fully anonymised dataset for 10 years, but the approval states that the dataset must be kept in a password protected university computer and not in a publicly accessible repository. These are detailed, highly sensitive in depth qualitative interviews with rich detail about patients’ lives and are therefore very difficult to truly render anonymous.

Participants consented to the publication of verbatim quotes and to the secondary analysis of the data for 10 years but did not consent to the full transcripts being publicly available.

We would therefore ask for our data to be made available through reasonable request to the corresponding author.

7. Please amend your list of authors on the manuscript to ensure that each author is linked to an affiliation. Authors’ affiliations should reflect the institution where the work was done (if authors moved subsequently, you can also list the new affiliation stating “current affiliation:….” as necessary).

All authors now have affiliations.

8. If the reviewer comments include a recommendation to cite specific previously published works, please review and evaluate these publications to determine whether they are relevant and should be cited. There is no requirement to cite these works unless the editor has indicated otherwise.

We were not asked by any reviewer to cite any additional works.

On behalf of the authors, I would like to thank you for the opportunity to amend and resubmit our work. I look forward to your response.

Yours truly,

Dr Catrin H Jones

1. Jones C, Cairns R, Walker H, Welsh S, Edgar B, Stevenson K, et al. Exploration of treatment burden through examination of workload and patient capacity during transition onto kidney replacement therapy: a systematic review of qualitative research. BMC medicine. 2025. p. 61. doi:10.1186/s12916-025-03904-7 PubMed PMID: 39901233.

2. May CR, Eton DT, Boehmer K, Gallacher K, Hunt K, MacDonald S, et al. Rethinking the patient: Using Burden of Treatment Theory to understand the changing dynamics of illness. BMC Health Serv Res. 2014 Jun 26;14(1). doi:10.1186/1472-6963-14-281 PubMed PMID: 24969758.

3. Kyle J, Skleparis D, Mair FS, Gallacher KI. What helps and hinders the provision of healthcare that minimises treatment burden and maximises patient capacity? A qualitative study of stroke health professional perspectives. BMJ Open. 2020 Mar 18;10(3). doi:10.1136/bmjopen-2019-034113 PubMed PMID: 32193265.

4. Gallacher KI, May CR, Langhorne P, Mair FS. A conceptual model of treatment burden and patient capacity in stroke. BMC Fam Pract. 2018 Jan 9;19(1). doi:10.1186/s12875-017-0691-4 PubMed PMID: 29316892.

5. Gallacher K, May CR, Montori VM, Mair FS. Understanding patients’ experiences of treatment burden in chronic heart failure using normalization process theory. Ann Fam Med. 2011;9(3):235–43. doi:10.1370/afm.1249 PubMed PMID: 21555751.

6. Adam R, Nair R, Duncan LF, Yeoh E, Chan J, Vilenskaya V, et al. Treatment burden in individuals living with and beyond cancer: A systematic review of qualitative literature. PLoS One. 2023 May 1;18(5 May). doi:10.1371/journal.pone.0286308 PubMed PMID: 37228101.

7. Gallacher K, Morrison D, Jani B, Macdonald S, May CR, Montori VM, et al. Uncovering Treatment Burden as a Key Concept for Stroke Care: A Systematic Review of Qualitative Research. PLoS Medicine. 2013. doi:10.1371/journal.pmed.1001473 PubMed PMID: 23824703.

8. Boehmer KR, Gallacher KI, Lippiett KA, Mair FS, May CR, Montori VM. Minimally Disruptive Medicine: Progress 10 Years Later. Mayo Clinic Proceedings. Elsevier Ltd; 2022. p. 210–20. doi:10.1016/j.mayocp.2021.09.003 PubMed PMID: 35120690.

9. May CR, Albers B, Bracher M, Finch TL, Gilbert A, Girling M, et al. Translational framework for implementation evaluation and research: a normalisation process theory coding manual for qualitative research and instrument development. Implementation Science. 2022 Dec 1;17(1). doi:10.1186/s13012-022-01191-x PubMed PMID: 35193611.

10. Malterud K, Siersma VD, Guassora AD. Sample Size in Qualitative Interview Studies: Guided by Information Power. Qual Health Res. 2016 Nov 1;26(13):1753–60. doi:10.1177/1049732315617444 PubMed PMID: 26613970.

11. Age disparities in patients with kidney failure in England and Wales A UK Kidney Association Disparities Sub-report Chair of the UK Kidney Association Patient Council-Sue Lyon.

12. Braun Virginia, Clarke Victoria. Successful qualitative research. 2013;382.

Attachment Submitted filename: PLOS One revisions letter version 3.docx

# Peer review: Decision Letter 1

**DOI:** 10.1371/journal.pone.0360290.r004

**Authors:** Wisit Kaewput

7 Jul 2026

Dear Dr. Jones,

Thank you for submitting your manuscript to PLOS ONE. After careful consideration, we feel that it has merit but does not fully meet PLOS ONE’s publication criteria as it currently stands. Therefore, we invite you to submit a revised version of the manuscript that addresses the points raised during the review process.

==============================

**ACADEMIC EDITOR:**

Thank you for the thoughtful and comprehensive revision. The manuscript is considerably improved. The revised framing of healthcare system processes as a prominent and potentially modifiable contributor to treatment burden is more balanced and better supported by the data. The expanded description of the sampling strategy, information power, analytic process, and reflexivity also improves the transparency and credibility of the study. The added longitudinal vignettes and figure are particularly helpful in illustrating how treatment burden evolves over time.

I have only a few minor comments for further improvement:

1. The abstract would be strengthened by explicitly stating the longitudinal nature of the study and the total number of interviews conducted. For example, the Methods and Findings section could state that 30 participants were interviewed across up to three timepoints, generating 76 interviews. This would better highlight one of the study’s key strengths.

2. The manuscript now provides a useful explanation of information power. However, because some readers may expect discussion of thematic saturation, it would be helpful to state explicitly that information power, rather than thematic saturation, was used to guide sample sufficiency. This would avoid ambiguity and align the methods more clearly with contemporary qualitative methodology.

3. If detailed screening, eligibility, or refusal numbers were not collected, this should be acknowledged clearly as a limitation. The explanation that this was an interpretive qualitative study is reasonable, but a brief acknowledgement that the absence of these data limits assessment of recruitment flow and possible selection bias would strengthen transparency.

4. The expanded analysis section is much improved. The authors may consider adding one or two sentences clarifying how analytic disagreements or interpretive uncertainties were handled during coding clinics, and whether negative or divergent cases were actively considered when developing the final themes. This would further strengthen confidence in the analytic process.

5. The revised manuscript appropriately acknowledges heterogeneity across modality pathways. Please ensure that any remaining statements comparing transplant, haemodialysis, and peritoneal dialysis are phrased cautiously, given the small and clinically heterogeneous subgroups.

6. A final careful copyedit is recommended to address minor grammatical, formatting, and spacing issues. In the abstract, the phrase “Using a framework analysis informed by…” could be revised to “We used framework analysis informed by…” for grammatical clarity. Please also ensure consistent spelling and hyphenation of terms such as “KRT transition-related workload,” “healthcare workload,” and “healthcare system.”

==============================

Please submit your revised manuscript by Aug 21 2026 11:59PM. If you will need more time than this to complete your revisions, please reply to this message or contact the journal office at plosone@plos.org. When you're ready to submit your revision, log on to https://www.editorialmanager.com/pone/ and select the 'Submissions Needing Revision' folder to locate your manuscript file.

- A letter that responds to each point raised by the academic editor and reviewer(s). You should upload this letter as a separate file labeled 'Response to Reviewers'.
- A marked-up copy of your manuscript that highlights changes made to the original version. You should upload this as a separate file labeled 'Revised Manuscript with Track Changes'.
- An unmarked version of your revised paper without tracked changes. You should upload this as a separate file labeled 'Manuscript'.

If you would like to make changes to your financial disclosure, please include your updated statement in your cover letter. Guidelines for resubmitting your figure files are available below the reviewer comments at the end of this letter.

If applicable, we recommend that you deposit your laboratory protocols in protocols.io to enhance the reproducibility of your results. Protocols.io assigns your protocol its own identifier (DOI) so that it can be cited independently in the future. For instructions see: https://journals.plos.org/plosone/s/submission-guidelines#loc-laboratory-protocols. Additionally, PLOS ONE offers an option for publishing peer-reviewed Lab Protocol articles, which describe protocols hosted on protocols.io. Read more information on sharing protocols at https://plos.org/protocols?utm_medium=editorial-email&utm_source=authorletters&utm_campaign=protocols.

As the corresponding author, your ORCID iD is verified in the submission system and will appear in the published article. PLOS supports the use of ORCID, and we encourage all coauthors to register for an ORCID iD and use it as well. Please encourage your coauthors to verify their ORCID iD within the submission system before final acceptance, as unverified ORCID iDs will not appear in the published article. *Only*  the individual author can complete the verification step; PLOS staff *cannot*  verify ORCID iDs on behalf of authors.

We look forward to receiving your revised manuscript.

Kind regards,

Wisit Kaewput, MD, FRCP(Lon), FRCP(Glasg), FASN, FAcadMEd

Academic Editor

PLOS One

Journal Requirements:

If the reviewer comments include a recommendation to cite specific previously published works, please review and evaluate these publications to determine whether they are relevant and should be cited. There is no requirement to cite these works unless the editor has indicated otherwise.

Please review your reference list to ensure that it is complete and correct. If you have cited papers that have been retracted, please include the rationale for doing so in the manuscript text, or remove these references and replace them with relevant current references. Any changes to the reference list should be mentioned in the rebuttal letter that accompanies your revised manuscript. If you need to cite a retracted article, indicate the article’s retracted status in the References list and also include a citation and full reference for the retraction notice.

Additional Editor Comments :

Thank you for the thoughtful and comprehensive revision. The manuscript is considerably improved. The revised framing of healthcare system processes as a prominent and potentially modifiable contributor to treatment burden is more balanced and better supported by the data. The expanded description of the sampling strategy, information power, analytic process, and reflexivity also improves the transparency and credibility of the study. The added longitudinal vignettes and figure are particularly helpful in illustrating how treatment burden evolves over time.

I have only a few minor comments for further improvement:

1. The abstract would be strengthened by explicitly stating the longitudinal nature of the study and the total number of interviews conducted. For example, the Methods and Findings section could state that 30 participants were interviewed across up to three timepoints, generating 76 interviews. This would better highlight one of the study’s key strengths.

2. The manuscript now provides a useful explanation of information power. However, because some readers may expect discussion of thematic saturation, it would be helpful to state explicitly that information power, rather than thematic saturation, was used to guide sample sufficiency. This would avoid ambiguity and align the methods more clearly with contemporary qualitative methodology.

3. If detailed screening, eligibility, or refusal numbers were not collected, this should be acknowledged clearly as a limitation. The explanation that this was an interpretive qualitative study is reasonable, but a brief acknowledgement that the absence of these data limits assessment of recruitment flow and possible selection bias would strengthen transparency.

4. The expanded analysis section is much improved. The authors may consider adding one or two sentences clarifying how analytic disagreements or interpretive uncertainties were handled during coding clinics, and whether negative or divergent cases were actively considered when developing the final themes. This would further strengthen confidence in the analytic process.

5. The revised manuscript appropriately acknowledges heterogeneity across modality pathways. Please ensure that any remaining statements comparing transplant, haemodialysis, and peritoneal dialysis are phrased cautiously, given the small and clinically heterogeneous subgroups.

6. A final careful copyedit is recommended to address minor grammatical, formatting, and spacing issues. In the abstract, the phrase “Using a framework analysis informed by…” could be revised to “We used framework analysis informed by…” for grammatical clarity. Please also ensure consistent spelling and hyphenation of terms such as “KRT transition-related workload,” “healthcare workload,” and “healthcare system.”

[Note: HTML markup is below. Please do not edit.]

Reviewers' comments:

Reviewer’s Responses to Questions

**Comments to the Author**

Reviewer #3: All comments have been addressed

**********

2. Is the manuscript technically sound, and do the data support the conclusions?

Reviewer #3: Yes

**********

3. Has the statistical analysis been performed appropriately and rigorously?

Reviewer #3: Yes

**********

4. Have the authors made all data underlying the findings in their manuscript fully available?

The PLOS Data policy

Reviewer #3: Yes

**********

5. Is the manuscript presented in an intelligible fashion and written in standard English?

Reviewer #3: Yes

**********

Reviewer #3: Thank you for your thorough revision. The concerns regarding overreaching conclusions, ethnic diversity limitations, and the longitudinal presentation of findings have been addressed adequately. The trajectory figure is a valuable addition to the manuscript as well. All remaining limitations are acknowledged by the authors and require no further revision.

**********

what does this mean?). If published, this will include your full peer review and any attached files.

If you choose “no”, your identity will remain anonymous but your review may still be made public.

**Do you want your identity to be public for this peer review?**  For information about this choice, including consent withdrawal, please see our Privacy Policy

Reviewer #3: **Yes:**  Omar Alfailakawi

**********

[NOTE: If reviewer comments were submitted as an attachment file, they will be attached to this email and accessible via the submission site. Please log into your account, locate the manuscript record, and check for the action link "View Attachments". If this link does not appear, there are no attachment files.]

To ensure your figures meet our technical requirements, please review our figure guidelines: https://journals.plos.org/plosone/s/figures

You may also use PLOS’s free figure tool, NAAS, to help you prepare publication quality figures: https://journals.plos.org/plosone/s/figures#loc-tools-for-figure-preparation.

NAAS will assess whether your figures meet our technical requirements by comparing each figure against our figure specifications.

# Author comment: Author response to Decision Letter 2

**DOI:** 10.1371/journal.pone.0360290.r005

6 Aug 2026

We would like to thank the academic editor for their thoughtful comments, and have made the following alterations to the manuscript as outlined below.

1. The abstract would be strengthened by explicitly stating the longitudinal nature of the study and the total number of interviews conducted. For example, the Methods and Findings section could state that 30 participants were interviewed across up to three timepoints, generating 76 interviews. This would better highlight one of the study’s key strengths.

Thank you for your suggestion. Line 21 has now been amended to read “Serial in-depth qualitative interviews were conducted with 30 participants across up to three time points, generating 76 interviews.”.

2. The manuscript now provides a useful explanation of information power. However, because some readers may expect discussion of thematic saturation, it would be helpful to state explicitly that information power, rather than thematic saturation, was used to guide sample sufficiency. This would avoid ambiguity and align the methods more clearly with contemporary qualitative methodology.

Thank you for your comment. We have added the following passage at lines 102-108 to expand on why information power was chosen as our approach to sample sufficiency:

“We elected to use information power to inform our sample size rather than thematic saturation as information power considers whether the data collected is sufficiently rich to answer the research question, rather than focus on the absence of new themes emerging. Thematic saturation has been criticised for being ambiguous in what truly represents saturation, and for encouraging coarse coding and broad themes in order to achieve so-called data saturation rather than deep reflexive and interpretative engagement with the data(7).”

This includes an additional reference to the paper “To saturate or not to saturate? Questioning data saturation as an useful concept for thematic analysis and sample-size rationales” by Braun and Clarke.

3. If detailed screening, eligibility, or refusal numbers were not collected, this should be acknowledged clearly as a limitation. The explanation that this was an interpretive qualitative study is reasonable, but a brief acknowledgement that the absence of these data limits assessment of recruitment flow and possible selection bias would strengthen transparency.

Thank you for your recommendation. The following passage has been added at lines 657-663 to recognise although we would maintain as authors that our decision not to collect detailed screening and eligibility numbers is epistemologically and methodologically sound as the sampling approach was purposive rather than probability based, the absence of these data limits the assessment of the recruitment process and limits the readers’ ability to scrutinise for selection bias:

“During the recruitment process we did not collect detailed screening, eligibility, or refusal numbers. The reason for this is that our epistemiological stance for this work is interpretivists and that has informed our purposive sampling strategy. We aimed to recruit a rich sample that was representative of the range of experiences rather than a probability based statistically representative sample as would be sought in studies with a positivist approach. However, the absence of these data limits the assessment of recruitment flow or potential selection bias.”

4. The expanded analysis section is much improved. The authors may consider adding one or two sentences clarifying how analytic disagreements or interpretive uncertainties were handled during coding clinics, and whether negative or divergent cases were actively considered when developing the final themes. This would further strengthen confidence in the analytic process.

Thank you for your comment. The reality is that the authors who conducted the coding clinics did not diverge greatly in their application of the coding framework and the analytic disagreement was minor. This has been reflected in the addition of this following sentence at 132-134:

“Analytic disagreements between the two authors were rare, and were resolved through discussion.”

Perhaps unsurprisingly considering how narrow and defined our phenomenon of interest was, we found that although there was considerable heterogeneity in our population in terms of KRT modality, life circumstances, clinical progression, the severity and the evolution over time of their treatment burden there were no negative cases (i.e. participants that did not experience any burden) or cases that diverged greatly in the nature rather than the extent of their burden. As there were no cases where our themes did not fit, we did not have any negative or highly divergent cases to consider in the analysis. The following has been added at line 147-148 to reflect this:

“There were no negative or divergent cases that required active consideration during the analysis process.”

5. The revised manuscript appropriately acknowledges heterogeneity across modality pathways. Please ensure that any remaining statements comparing transplant, haemodialysis, and peritoneal dialysis are phrased cautiously, given the small and clinically heterogeneous subgroups.

Thank you for your recommendation. The following alterations have been made to phrase conclusions about subgroups more cautiously.

Line 558-560 now reads:

“Thirdly, successful transplantation can have a transformative effect on burden, offering the possibility of a much lower healthcare workload and increased capacity due to improved physical health.”

Line 693-695 now reads :

“Secondly, for eligible patients, successful kidney transplantation could represent the treatment pathway that offers the lowest workload and burden, and interventions that improve access to transplantation are key to reducing treatment burden”

6. A final careful copyedit is recommended to address minor grammatical, formatting, and spacing issues. In the abstract, the phrase “Using a framework analysis informed by…” could be revised to “We used framework analysis informed by…” for grammatical clarity. Please also ensure consistent spelling and hyphenation of terms such as “KRT transition-related workload,” “healthcare workload,” and “healthcare system.”

A final copyedit has been completed with several minor grammatical, formatting and spacing alterations.

# Editor report: Decision Letter 2

**DOI:** 10.1371/journal.pone.0360290.r006

**Authors:** Wisit Kaewput

7 Aug 2026

Dear Dr. Jones,

Thank you for submitting your manuscript to PLOS ONE. After careful consideration, we feel that it has merit but does not fully meet PLOS ONE’s publication criteria as it currently stands. Therefore, we invite you to submit a revised version of the manuscript that addresses the points raised during the review process.

Please submit your revised manuscript by Sep 21 2026 11:59PM. If you will need more time than this to complete your revisions, please reply to this message or contact the journal office at plosone@plos.org. When you're ready to submit your revision, log on to https://www.editorialmanager.com/pone/ and select the 'Submissions Needing Revision' folder to locate your manuscript file.

- A letter that responds to each point raised by the academic editor and reviewer(s). You should upload this letter as a separate file labeled 'Response to Reviewers'.
- A marked-up copy of your manuscript that highlights changes made to the original version. You should upload this as a separate file labeled 'Revised Manuscript with Track Changes'.
- An unmarked version of your revised paper without tracked changes. You should upload this as a separate file labeled 'Manuscript'.

If you would like to make changes to your financial disclosure, please include your updated statement in your cover letter. Guidelines for resubmitting your figure files are available below the reviewer comments at the end of this letter.

If applicable, we recommend that you deposit your laboratory protocols in protocols.io to enhance the reproducibility of your results. Protocols.io assigns your protocol its own identifier (DOI) so that it can be cited independently in the future. For instructions see: https://journals.plos.org/plosone/s/submission-guidelines#loc-laboratory-protocols. Additionally, PLOS ONE offers an option for publishing peer-reviewed Lab Protocol articles, which describe protocols hosted on protocols.io. Read more information on sharing protocols at https://plos.org/protocols?utm_medium=editorial-email&utm_source=authorletters&utm_campaign=protocols.

As the corresponding author, your ORCID iD is verified in the submission system and will appear in the published article. PLOS supports the use of ORCID, and we encourage all coauthors to register for an ORCID iD and use it as well. Please encourage your coauthors to verify their ORCID iD within the submission system before final acceptance, as unverified ORCID iDs will not appear in the published article. *Only*  the individual author can complete the verification step; PLOS staff *cannot*  verify ORCID iDs on behalf of authors.

We look forward to receiving your revised manuscript.

Kind regards,

Wisit Kaewput, MD, FRCP(London), FRCP(Glasgow), FRCP(Thailand), FASN, FAcadMEd

Academic Editor

PLOS One

Journal Requirements:

1. If the reviewer comments include a recommendation to cite specific previously published works, please review and evaluate these publications to determine whether they are relevant and should be cited. There is no requirement to cite these works unless the editor has indicated otherwise.

2. Please review your reference list to ensure that it is complete and correct. If you have cited papers that have been retracted, please include the rationale for doing so in the manuscript text, or remove these references and replace them with relevant current references. Any changes to the reference list should be mentioned in the rebuttal letter that accompanies your revised manuscript. If you need to cite a retracted article, indicate the article’s retracted status in the References list and also include a citation and full reference for the retraction notice.

Additional Editor Comments:

The manuscript has been substantially improved, and the previous scientific concerns have been satisfactorily addressed. No further external peer review is required. Before acceptance, I request only a final editorial revision to ensure compliance with PLOS ONE’s Data Availability Policy for qualitative participant data, to correct the remaining grammatical and formatting issues, and to slightly temper the concluding statement regarding transplantation given the small and heterogeneous modality subgroups. No additional analyses are required.

[Note: HTML markup is below. Please do not edit.]

[NOTE: If reviewer comments were submitted as an attachment file, they will be attached to this email and accessible via the submission site. Please log into your account, locate the manuscript record, and check for the action link "View Attachments". If this link does not appear, there are no attachment files.]

To ensure your figures meet our technical requirements, please review our figure guidelines: https://journals.plos.org/plosone/s/figures

You may also use PLOS’s free figure tool, NAAS, to help you prepare publication quality figures: https://journals.plos.org/plosone/s/figures#loc-tools-for-figure-preparation.

NAAS will assess whether your figures meet our technical requirements by comparing each figure against our figure specifications.

# Author comment: Author response to Decision Letter 3

**DOI:** 10.1371/journal.pone.0360290.r007

9 Sep 2026

I have now included a cover letter, an unmarked manuscript and a manuscript with mark up

Attachment Submitted filename: plos one response to reviewers.docx

# Editor report: Decision Letter 3

**DOI:** 10.1371/journal.pone.0360290.r008

**Authors:** Wisit Kaewput

24 Sep 2026

Exploration of treatment burden during transition onto kidney replacement therapy: a longitudinal qualitative study

PONE-D-26-04817R3

Dear Dr. Jones,

We’re pleased to inform you that your manuscript has been judged scientifically suitable for publication and will be formally accepted for publication once it meets all outstanding technical requirements.

Within one week, you’ll receive an e-mail detailing the required amendments. When these have been addressed, you’ll receive a formal acceptance letter and your manuscript will be scheduled for publication.

An invoice will be generated when your article is formally accepted. Please note, if your institution has a publishing partnership with PLOS and your article meets the relevant criteria, all or part of your publication costs will be covered. Please make sure your user information is up-to-date by logging into Editorial Manager at Editorial Manager® and clicking the ‘Update My Information' link at the top of the page. For questions related to billing, please contact billing support.

If your institution or institutions have a press office, please notify them about your upcoming paper to help maximize its impact. If they’ll be preparing press materials, please inform our press team as soon as possible -- no later than 48 hours after receiving the formal acceptance. Your manuscript will remain under strict press embargo until 2 pm Eastern Time on the date of publication. For more information, please contact onepress@plos.org.

Kind regards,

Wisit Kaewput, MD, FRCP(London), FRCP(Glasgow), FRCP(Thailand), FASN, FAcadMEd

Academic Editor

PLOS One

Additional Editor Comments (optional):

Accept as is.

Reviewers' comments:

# Editor report: Acceptance letter

**DOI:** 10.1371/journal.pone.0360290.r009

**Authors:** Wisit Kaewput

PONE-D-26-04817R3

PLOS One

Dear Dr. Jones,

I'm pleased to inform you that your manuscript has been deemed suitable for publication in PLOS One. Congratulations! Your manuscript is now being handed over to our production team.

At this stage, our production department will prepare your paper for publication. This includes ensuring the following:

* All references, tables, and figures are properly cited

* All relevant supporting information is included in the manuscript submission,

* There are no issues that prevent the paper from being properly typeset

You will receive further instructions from the production team, including instructions on how to review your proof when it is ready. Please keep in mind that we are working through a large volume of accepted articles, so please give us a few days to review your paper and let you know the next and final steps.

Lastly, if your institution or institutions have a press office, please let them know about your upcoming paper now to help maximize its impact. If they'll be preparing press materials, please inform our press team within the next 48 hours. Your manuscript will remain under strict press embargo until 2 pm Eastern Time on the date of publication. For more information, please contact onepress@plos.org.

You will receive an invoice from PLOS for your publication fee after your manuscript has reached the completed accept phase. If you receive an email requesting payment before acceptance or for any other service, this may be a phishing scheme. Learn how to identify phishing emails and protect your accounts at https://explore.plos.org/phishing.

If we can help with anything else, please email us at customercare@plos.org.

Thank you for submitting your work to PLOS One and supporting open access.

Kind regards,

PLOS One Editorial Office Staff

on behalf of

Dr. Wisit Kaewput

Academic Editor

PLOS One
